# Graphene and Its Derivatives: Synthesis and Application in the Electrochemical Detection of Analytes in Sweat

**DOI:** 10.3390/bios12100910

**Published:** 2022-10-21

**Authors:** Anoop Singh, Aamir Ahmed, Asha Sharma, Sandeep Arya

**Affiliations:** Department of Physics, University of Jammu, Jammu 180006, India

**Keywords:** wearable, sensors, graphene, invasive, health monitoring, non-invasive

## Abstract

Wearable sensors and invasive devices have been studied extensively in recent years as the demand for real-time human healthcare applications and seamless human–machine interaction has risen exponentially. An explosion in sensor research throughout the globe has been ignited by the unique features such as thermal, electrical, and mechanical properties of graphene. This includes wearable sensors and implants, which can detect a wide range of data, including body temperature, pulse oxygenation, blood pressure, glucose, and the other analytes present in sweat. Graphene-based sensors for real-time human health monitoring are also being developed. This review is a comprehensive discussion about the properties of graphene, routes to its synthesis, derivatives of graphene, etc. Moreover, the basic features of a biosensor along with the chemistry of sweat are also discussed in detail. The review mainly focusses on the graphene and its derivative-based wearable sensors for the detection of analytes in sweat. Graphene-based sensors for health monitoring will be examined and explained in this study as an overview of the most current innovations in sensor designs, sensing processes, technological advancements, sensor system components, and potential hurdles. The future holds great opportunities for the development of efficient and advanced graphene-based sensors for the detection of analytes in sweat.

## 1. Introduction

The healthcare system is facing rising prices and difficulties as the world population grows fast and human life expectancy rises dramatically [1,2], necessitating governments to discover realistic ways to provide basic medical treatment without raising healthcare costs [3]. Early detection and diagnosis are possible using preventive and customised medicine techniques [4], which alter with health condition. Additionally, disease risk may be forecasted and employed to overcome obstacles by boosting the cure rate and survival of a population at risk, while reducing total treatment costs [5,6]. Health monitoring systems can comprehensively assess health conditions by tracking critical signs and biomarkers on a regular or continuous basis, which can significantly benefit diagnosis and disease treatment, as well as postoperative rehabilitation, reducing the burden on medical systems and improving quality of life [7].

Sensors, especially wearable and implanted sensors, are key components of health monitoring systems and the interface to the human body because they can detect and analyse numerous analytes or signals with high sensitivity and specificity [5]. Standard stiff silicon-based sensors cannot match the skin’s mechanical flexibility, which means intrusive sensors must include flexible mechanical components [8]. Stability, biocompatibility, comfort, dependability, costs, miniaturisation, convenience and biofouling should all be evaluated or even bargained for location-unlimited, multifunctional, real-time, long-term, widespread, unobtrusive, and economical health monitoring [9]. Furthermore, since these sensors can gather a significant quantity of data, contemporary outstanding management of data and analysis approaches, such as Big Data [10,11,12] and machine learning [13], are utilised in data handling and effective information mining [14,15]. As a result, the security and privacy of personal data must be appropriately ensured.

**Figure 1:**
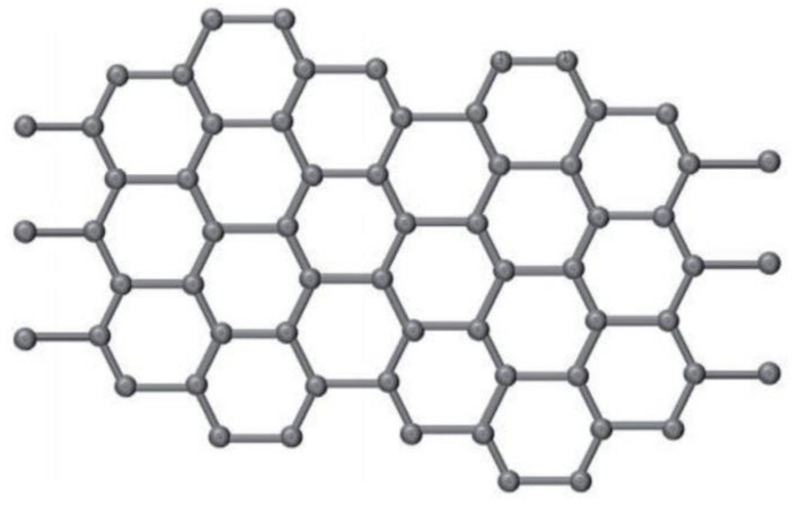
Structure of graphene. Reproduced from [35] under common creative 3.0. license. (Copyright 2020, IntechOpen, London, UK).

Wearable and implanted sensors for health monitoring are being developed, and graphene is a desirable 2D material because of its amazing multimodal features, i.e., ultrahigh carrier mobility [16,17], excellent electrical conductivity [18,19], large specific surface area [20], superior thermal conductivity, high optical transmittance [21], high Young’s modulus [22], and outstanding mechanical flexibility [23]. The performance diversity of graphene allows for the creation of a variety of multifunctional sensors. The following are some of the benefits of graphene for sensors: The first argument is that since graphene layers have a large specific surface area and an atomic thickness that allows complete carbon atoms to come into direct contact with analytes, graphene-based sensors offer higher sensitivity than silicon-based sensors [24]. Graphene-based sensors can also accomplish conformal, special connection with required organs such as the skin [25], brain [26], and eyes [27], which are critical for attaining high quality signals without irritation, motion artefacts, or contamination [28]. This is because of graphene’s flexibility in mechanical strength and fine thickness. Graphene’s outstanding electrical conductivity and optical transparency also make it a perfect material for seeing bio-tissue in clear images with no distortions in visual appearance [29]. As a result, electrophysiological signals may be recorded with high SNR (ratio of signal-to-noise) using techniques such as conformal integration [30]. Furthermore, due to graphene’s superior performance in biosensors, such as wide potential window, ease of functionalization, its large specific surface area, and high rate for transference of electrons, receptors such as deoxyribonucleic acid, antibodies and enzymes can be efficiently immobilised on its surface [31]. More information on the characteristics, production, characterisation, and various uses of graphene and its derivatives may be found in prior review studies [32,33,34], which were not included in this study owing to space constraints. The structure of graphene is shown in Figure 1 [35].

The necessity and significance of monitoring systems and the advantages of graphene in sensing devices and its non-toxicity are explored. In the next part, we examine the most recent sensors based on graphene, covering non-invasive and invasive condition monitoring, as well as their distinctive architectures, detecting methodology, and pace of innovation. Various sensor system components are also shown. Graphene-based sensing systems are also examined in terms of their potential drawbacks and advantages in the future.

## 2. Techniques for the Production of Graphene

**Figure 2:**
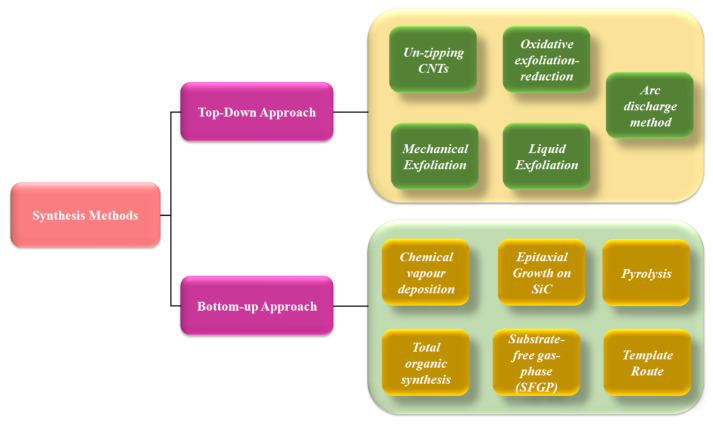
Synthesis approach for graphene.

To be effectively used in agency, graphene must be manufactured at a price comparable to that of currently available materials. It is a significant problem to create production methods that are cost efficient, highly dependable, and scalable, as well as that produce high yields and quality products [36,37]. As a result, the following description of synthesis techniques concentrates mostly on these characteristics. Graphene’s production methods and comprehensive characterisation have been described elsewhere in the literature [38,39]. The top-down (destruction) and bottom-up (construction) techniques for graphene production are the two major methodologies [40]. The complete synthesis approaches are shown in Figure 2.

### 2.1. Top-Down Methods

The top-down approach is defined as a tactic that concentrates on the attack of powdered raw graphite. Eventually, the attack will split its layers and create graphene sheets. Chemical synthesis and mechanical or chemical exfoliation, for example, are classified as top-down strategies [41]. Some of the commonly used top-down approaches for graphene synthesis are discussed below.

#### 2.1.1. Oxidative Exfoliation Reduction

**Figure 3:**
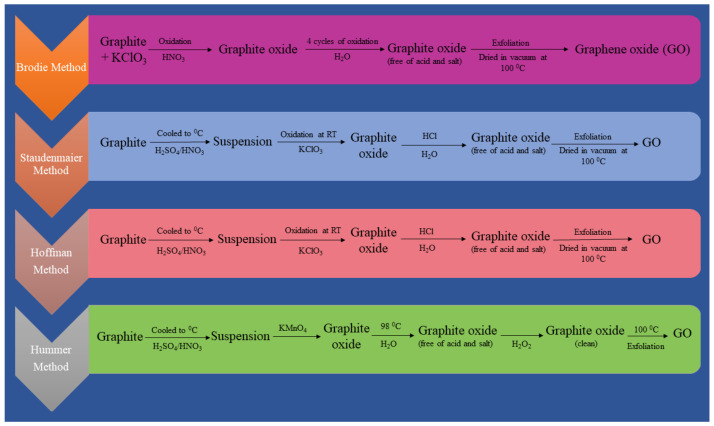
Graphite oxidation route schemes.

The majority of graphene oxide (GO) is produced via oxidative graphite exfoliation followed by reduction to reduced graphene oxide (rGO) or graphene sheets. Staudenmaier, Brodie, Hummers and Hofmann are four of the most popular ways for GO production [42,43]. The reaction sequences of these techniques that operate at temperatures below 100 °C are shown in Figure 3. To keep manufacturing costs low, the synthesis temperature should be maintained as low. These techniques, however, produce poisonous gases such and dinitrogen tetroxide (N_2_O_4_) and nitrogen dioxide (NO_2_) [44]. As a result, while scaling up a process, environmental costs and process safety must be taken into account. The Hummers technique is now extensively utilised for GO synthesis since it is a very quick and safe approach. Furthermore, by using sodium nitrate (NaNO_3_) and potassium permanganate (KMnO_4_) instead of nitric acid (HNO_3_) and potassium perchlorate (KClO_4_), it does not produce harmful gases such as ClO_2_ (chlorine dioxide) or acidic fog [45]. Modifications to the Hummers technique have resulted in a more environmentally friendly way of developing GO over time [46]. For example, the enhanced Hummers technique no longer uses NaNO_3_ to synthesise GO, lowering manufacturing costs and environmental risks [45]. Significantly with the addition of graphite intercalation components, graphite’s oxidation enhances the separation between layers of graphite [47]. Carbon sheet displacement reduces van der Waals forces between layers, which may lead to well-distributed single-, bi-, and few-layer GO formations in suitable solvents [47]. Owing to the existence of oxygenous functional groups, GO is extremely hydrophilic and can disperse in a variety of fluids, including ethylene glycol, tetrahydrofuran (THF), water, and N-methyl-2 pyrrolidone (NMP) [48]. Because GO’s sp^2^ bonding is disrupted, reduction techniques are frequently used to rebuild its honeycomb lattice. Electrochemical, thermal and chemical techniques are the most common ways to reduce GO. During reduction, the majority of the functional groups that contain oxygen in GO, such as carboxylic, hydroxyl and carbonyl, are removed. However, it is still impossible to completely reduce GO to synthesize pure graphene [49]. The resultant rGO is often quite similar to pure graphene, albeit with certain flaws and size variations. Reductant variety and operational parameters such as voltage, pressure, and temperature and reduction duration influence the quality of rGO. Additionally, the C/O ratio indicates if a reductant is suitable for reduction of GO [50]. A high C/O ratio leads to a high level of deoxygenation, resulting in better rGO quality. Hydrazine (N_2_H_4_) is frequently used to decrease the oxygen content of GO in chemical reduction. Furthermore, N_2_H_4_ is both costly and poisonous, which limit its use on a wide scale. As a result, a more environmentally friendly method was developed to replace the hazardous N_2_H_4_ utilised in GO reduction [51]. Proteins, microbes, plant extracts, amino acid, metal–alkaline, metal–acid, reagents (nitrogen, sulphur, oxygen), hydrohalic acid, aluminium hydrides, borohydrides, and hormones were among the reduction agents employed [50]. Using sodium borohydride (6.9) [52], caffeic acid (7.15) [53], thionyl chloride (8.48) [54], benzyl alcohol (30) [55], zinc/hydrochloric acid (33.5) [56], and baker’s yeast (5.9) [57], the chemical reduction produced high C/O, but issues of safety, additional cost of chemical, pollution, and a long synthesis time must be measured throughout course scale-up. As a result, researchers are actively looking into alternate ways of GO reduction, such as hydrothermal and thermal reduction [58,59]. The emission of greenhouse gases and the high temperature related with thermal reduction are the two most significant problems. Hydrothermal reduction, however, has been demonstrated to convert GO to rGO using less energy [60]. Because of its environmental friendliness, economic effectiveness, fast reduction and ease of application, electrochemical GO reduction has attracted much interest. More significantly, as compared to chemical reduction, it uses less hazardous reductants. Microwave, sonochemical, photocatalytic, laser, photothermal and plasma treatment are some of the various GO reduction methods described in the literature [61]. The manufacture of GO by oxidative exfoliation of graphite and subsequent reduction to rGO has a reasonable cost and high yield. However, owing to van der Waals attraction, the rGO has a limited surface area, irreversible sheet restacking, low solubility and weak electrical conductivity [62]. The process of synthesis is rife with unknowns, such as batch-to-batch repeatability, chemical composition changes, and the formation of a permanent flaw during the oxidation stage [46]. Nonetheless, the benefits of great scalability and cheap operating costs exceed the method’s drawbacks.

#### 2.1.2. Arc Discharge Method

**Figure 4:**
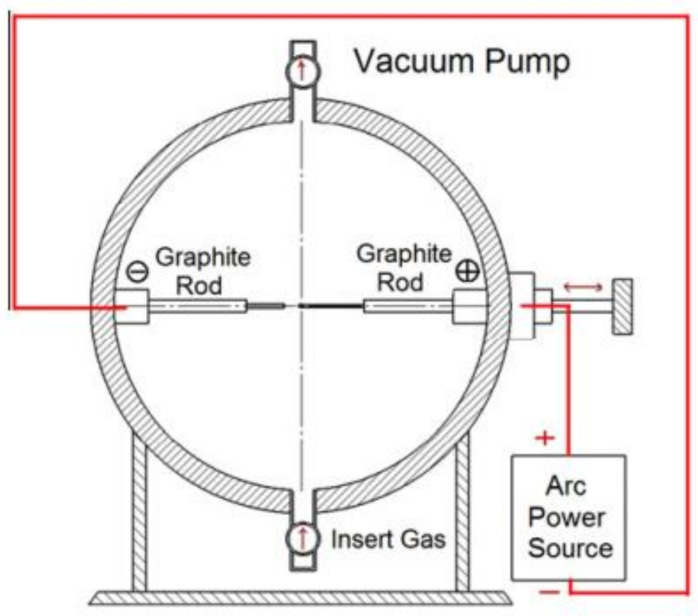
A schematic diagram of the DC arc discharge apparatus. (Reproduced from [70] with permission. Copyright 2012, Elsevier, Amsterdam, The Netherlands).

This method has been used to make graphene that is pure, B-doped, and N-doped. The environment in which graphene is synthesised has a significant impact on the final product. The technique was originally used to manufacture graphene by Kratschmer et al. [63]. This method has enabled the production of 2–4 layers graphene [64]. Several carbon-based nanomaterials, such as fullerenes and carbon nanotubes, have been successfully produced utilising the arc discharge technique [65,66]. Hydrogen arc discharge produced graphene sheets (petal-like) on the cathode’s surface, according to the study’s findings. Two to four layers of graphene were successfully fabricated in a hydrogen–helium mixture environment in 2010 [67]. It has been discovered that various discharge atmospheres may induce distinct bonding, resulting in graphene with varied characteristics as produced. For this procedure, the electric arc discharge system or oven consists primarily of two electrodes and a steel chamber cooled by water. Cathode and anode terminals are made entirely of graphite rods. To ensure optimal operation, after generation, a steady current of 100–150 A is typically maintained at discharge. The kind of graphene created is determined by the various atmospheres of arc graphite rod evaporation in which it is produced. The most frequent conditions in which graphene is generated in this process are H_2_, ammonia–helium and air. The discharge atmosphere is an ammonia–helium combination, which produces N-doped graphene sheets. O-C=O and C-O bonds are added into the mixture of CO_2_-He to manufacture graphene sheets with excellent dispersity and electrical conductivity. There is a plasma discharge when the rods of graphite are brought close together. Through the rotation of the cathode at a fixed distance of around 1–2 mm from the anode, graphene may be generated. Discharged soot is collected under ambient circumstances after it has been disbursed. It is B-doped graphene that is created when an arc discharge is carried out in an environment of hydrogen and andiborane and ammonia combination [68], while N-doped graphene is produced when an ammonia–helium mixture is used [69]. To manufacture exceptionally crystalline graphene sheets, the arc discharge approach has been shown in various combinations of inert gas and H_2_. In this method, hydrogen graphene sheets are created by removing weakly connected carbon bonds, resulting in few-layer graphene sheets. The electrochemical and electrical performance of batteries (lithium-ion), which are common sources of power for portable electronic gadgets, may be improved using graphene sheets generated by the arc discharge technique. It is possible to improve the performance of batteries by incorporating graphene sheets into the cathode and anode. A DC H_2_ arc discharge utilising electrodes of pure graphite and various mixtures of gases may create large amounts of highly crystalline graphene. At 50 mA/g current density, studies have shown that the lithium-ion battery’s capacity of initial discharge may reach as high as 1332 mAh/g (where graphene sheets are employed to improve the electrodes). After 300 cycles, the retention incapacity is 323 mAh/g in this scenario [64]. The cyclic stability in the test cell is significantly enhanced with high crystallinity and sufficient thermal stability of FLG. The setup of the experiment for the arc discharge method is demonstrated in Figure 4.

#### 2.1.3. Liquid Exfoliation

**Figure 5:**
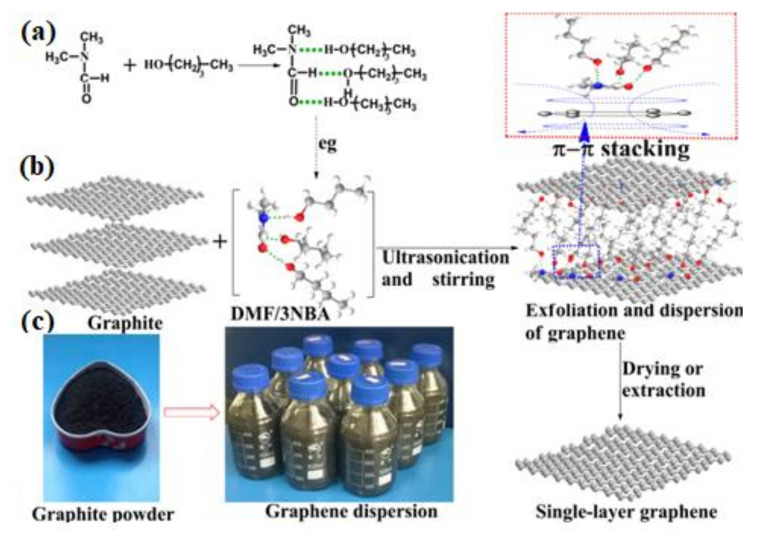
The FT-IR of solvents and schematic illustration of the exfoliation mechanism of graphene in the binary solvent system. (**a**) The development of bonding (hydrogen) between DMF and NBA molecules. (**b**) The exfoliation of graphite into few-layer graphene. (**c**) Graphite powder and graphene–DMF/3NBA dispersions. (Reproduced from [76] with permission. Copyright 2015, Elsevier, Amsterdam, The Netherlands).

Ultrasonic energy is utilised to make microcavitations and shatter graphite into small bits and thinner layers. To properly separate the graphite into distinct layers, a number of hours are required. Organic solvents such as DMEU (1,3-dimethyl-2-imidazolidinone), DMA(N,N-dimethylacetamide) and NMP (N-methylpyrrolidone) are used in the liquid exfoliation method [71,72,73,74,75]. Dispersion and exfoliation of graphite have lately been examined in surfactant/water solutions rather than in solvent of organic materials, with the stability described by the Derjaguin, Landau, Vervey, and Overbeek (DLVO) and Hamaker theory [71]. Exfoliated graphene with a surfactant coating may be stabilised as a colloid. Double layer surfactant is the result of the bounded-molecule surface producing a tail group. Due to the conflict between the repulsive solvent–graphene and interlayer van der Waals energy, exfoliation may be achieved. Figure 5 displays a graphite dispersion that has been blasted and exfoliated using ultrasonication and moderate centrifugation. In the transmission electron micrograph, an isolated single-layer graphene from the solution can be seen, with the distinctive hexagonal rings visible in the chosen electron diffraction region. Because of the easy intercalation, cavitation, and exfoliation procedures, this technique is extremely popular. During the manufacturing step, the exfoliated graphite layers exhibit a high throughput. Graphene sheets that have been subjected to an extended ultrasonic treatment, however, may be damaged, resulting in nano- and micro-sized contaminants. At a thickness of 10 to 100 nm, the exfoliated graphite layers lose their graphene-like electrical properties. Microwave radiation and annealing were employed after exfoliation to remove trapped solvent and air bubbles, resulting in a substantial increase in the volume of graphite layer (exfoliated). The creation of low-cost graphene electrode materials and sensors with huge surface areas and catalytic activity, especially electro, as well as mechanical reinforcement and optical limiters for polymer-based nanomaterials, has reached a significant milestone with the liquid exfoliation of graphene [75].

#### 2.1.4. Un-Zipping CNTs

This is a novel graphene production process that uses multi-wall nanotubes (MWNTs) as the precursors. The unzipping of MWNTs results in graphene. A successful first step was the intercalation of ammonia and lithium in an acidic medium to open the MWNT longitudinally. Exfoliation is then accomplished via rapid heat expansion. Nanoribbons or partly opened MWNTs, as well as graphene flakes, are the first products [77]. In a different method, the MWNTs are etched to open them and create graphene. It is possible to etch or unzip MWNTs in polymer films, resulting in partially opened nanoribbons or MWNTs. Exfoliation with H_2_SO_4_ in concentrated form followed by oxidation in stages with KMnO_4_ and, ultimately, NH_4_OH used for reduction are additional alternatives to a single-step chemical treatment technique [78].

#### 2.1.5. Mechanical Exfoliation

Monolayer graphene flake extraction on chosen substrates may be achieved via mechanical exfoliation. Graphene production has been reported for the first time using this technology. An application of this method in nanotechnology occurs when transverse or longitudinal stress is applied to the layered material surface. Due to weak van der Waals interactions, graphite is created when graphene layers are layered on top of each other. The bond energy and distance values for the interlayer data are 2 eV/nm^2^ and 3.34, respectively [79]. Mechanical cleaving, however, requires an external force of up to 300 nN/m^2^ in order to separate a single atomic layer from graphite [80]. Sheet stacking is the result of van der Waals forces acting perpendicularly on partially filled p orbitals on the sheet plane. With exfoliation, the reversal of stacking, there is a weak bonding and a huge vertical lattice gap that results. Because of this, there is a higher bonding and a smaller lattice gap on the hexagonal lattice plane [81]. As a result, graphene sheets of different thicknesses may be formed via mechanical exfoliation or methods such as peeling layers off graphitic substances such as natural graphite [82], mono-crystal graphite [83], and highly ordered pyrolytic graphite [84]. Agents such as electric field [85], ultrasonication [86], transfer printing technique [87], and scotch tape may be used to exfoliate.

The above-discussed methods are mostly used by the researchers. However, bottom-up methods are also reported in various works.

### 2.2. Bottom-Up Methods

The bottom-up approach, however, is described as a method that involves the use of carbonaceous gas to produce graphene. Pyrolysis, chemical vapour deposition (CVD), epitaxial growth, and other techniques utilising the bottom-up approach are illustrated below [41].

#### 2.2.1. Chemical Vapour Deposition

Chemical vapour deposition has shown to be a strong and efficient technique for producing large surface area graphene on a commercial scale for use in a variety of industrial applications. This technique employs a high temperature to break down activated carbons precursors on the substrate surfaces in order to produce high-quality graphene sheets [88]. Adsorption sites on the substrate surface are formed when precursors are pyrolytically degraded, allowing for the creation of homogeneous thin films on the substrate surface [89]. Carbon sources for CVD of graphene include PAHs (polycyclic aromatic hydrocarbons), benzene, C_2_H_2_ (ethylene), and CH_4_ (methane). This technique was utilised to create planar few-layer graphene for the first time [90]. The CVD method employs three distinct fundamental approaches: microwave-plasma-enhanced CVD, plasma-enhanced CVD and thermal CVD [91]. Other enhanced CVD methods have been discovered in an effort to dramatically decrease the process’s stimulation power, which minimises the need for high temperatures. The precursors breakdown at high temperatures to produce carbon atoms, which subsequently build a nanostructure of graphite from the fragmented atoms of carbon. A relatively higher temperature of approximately 250 °C is needed without a catalyst (metal) for CVD to produce a graphitic structure. Transition metals are often used to lower the growth temperature because they act as an efficient catalyst for rapidly converting hydrocarbons into graphitic materials [92]. Because of homogeneous nucleation and fewer structural flaws, graphene production on clean surfaces improves graphene quality [93]. For a diversity of industrial uses, the CVD technique of graphene production is dependable, practical, and cost-effective. Meanwhile, current research has focused on developing a more sustainable method for producing graphene in large quantities and of excellent quality using CVD at temperatures below 100 °C [94,95,96]. Portraying graphene as a suitable option for future devices (flexible electronic) has certain drawbacks that must be addressed. There are several challenges associated with CVD-based substrate graphene synthesis [96]. This contamination results in increases and severe flaws due to the migration of graphene from the metal catalyst surface to an insulating surface [97]. Graphene’s performance is often hampered by these flaws when it comes to practical applications [98]. Because of CVD’s high energy needs, scientists have been forced to search for alternative ways to achieve a more sustainable synthesis. Researchers must systematically investigate strategies to dramatically lower growth temperatures in order to cope with the difficulties of transferring graphene onto substrates (dielectric) and the related high energy consumption of CVD. Because of these concerns, low-temperature CVD for graphene straight deposition on substrates has emerged as a cost-effective and trusted technique for commercial graphene manufacturing [99]. Many factors, including type and thickness (thickness) of a CVD catalyst, amount of hydrocarbon, and CVD method, may influence CVD growth temperature. Catalyst plays an important function in CVD. In recent research, various metals include such as Cu, Co, Ru, Ni and Pt have been used to catalyse the graphene CVD process. MLG (multi-layered graphene) sheets are separated from the catalyst using a self-assembly method that uses C_60_ thin films (30 nm) instead of Ni films, allowing for direct characterisation of MLGs [100]. Cu and Ni catalysts, however, seem to be extensively employed, with Cu attracting a lot of interest because of its poor carbon solubility at high temperatures. In a CVD furnace, carbon precursors (such as ethylene, methane, etc.) are pyrolyzed and evaporated at 700–850 °C utilizing argon gas as the carrier to form single-layer graphene (SLG) to few-layer graphene (FLG) sheets on nickel substrates [101,102]. This is a novel method that has been hailed as low-cost and environmentally beneficial. Regulating the amount of graphene layers and folds as well as carbon solubility on Ni substrate and segregation kinetics are some of the drawbacks of this method of production. It has been shown that the graphene creation precipitation mechanism is not only based on a CVD-Ni system, but also on a process of catalysing a surface to accomplish a reaction. Due to Cu’s poor carbon solubility, it has been recognised as the best in terms of CVD performance. High temperatures cause a substantial amount of carbon in a catalyst to dissolve into its bulk, which inhibits its development [103]. Due of the poor solubility of carbon in Cu, SLG forms on the surface. Meanwhile, the highest graphene quality is obtained at temperatures near to the substrate’s (Cu) melting point. The cooling rate control in CVD is largely responsible for determining the resulting graphene sheet thickness. CVD also comprises precursor gas flow rate, the gas ratio, the reaction time, and the furnace temperature as significant factors [104]. SLG is favoured over MLG since it has the best graphene characteristics. At 100 °C, methane has been extensively utilised as a gaseous carbon precursor to manufacture homogenous SLG. Carbon adsorption on catalyst surfaces and dehydrogenation of methane generally take place [105]. The catalyst lowers the activation energy of the reaction, allowing it to continue at a quicker pace. It has been created by roll-to-roll manufacturing of graphene sheets utilising foil of copper as a continuous CVD of graphene [106]. To summarise, hydrocarbon sources with a particular chemical structure and low energy of activation allow for low-temperature synthesis [107]. However, because of the ring forms for carbon atoms, the usage of benzene and PAHs is recommended as the best method to reduce the activation energy for the creation of graphene. The development temperature of polycyclic aromatic hydrocarbons (PAHs) on a Cu substrate may be lowered by mixing in OPA (1-Octylphosphoric acid), an aliphatic hydrocarbon. Hydrocarbon materials, poly(methyl methacrylate) (PMMA), polystyrene and benzene as a precursor of liquid material have been explored for producing graphene on Cu foil at temperatures under 100 °C. Due to their low activation energy requirements, hydrocarbons greatly reduce the growing temperature on the surface of the catalyst.

#### 2.2.2. Epitaxial Growth on SiC

**Figure 6:**
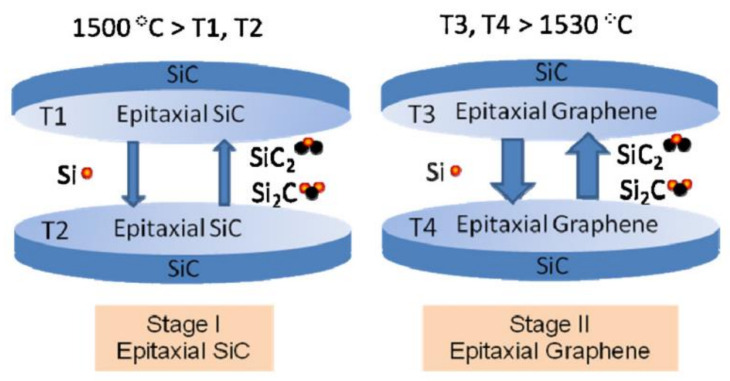
Schematic diagram of the two-step epitaxial graphene growth model. Reproduced from [118] under common creative.

Using an alternate method as shown in Figure 6, silicon carbide (SiC) is heated at 1250–1450 °C in ultrahigh vacuum (UHV). The carbon atoms remain on the SiC surface after the silicon ions sublimate. Under ideal conditions, carbon atoms self-organize into honeycomb structures. After that, the SiC-grown epitaxial graphene (EG) may be transferred to another substrate as free-standing graphene. To peel-off from SiC to a SiO_2_/Si wafer, EG uses a polyimide and thin gold layer. However, the resultant graphene sheet has many flaws and has a mobility of only 100 cm^2^/Vs [108]. Temperature control and the existence of defects or disorder in the hexagonal structure might be challenging to maintain with this technology. The electrical properties of EG have also been examined, and they reveal an opening of a band gap at 260 meV, at which time the Fermi level increases to 400 meV [109]. The width of the band gap is affected by the thickness of graphene. Many researchers have reported the graphene-SiC interface’s energy bandgap, growth process, and symmetry breakdown in great detail. The gap-opening phenomena between the two bands may have two potential causes. One option is to break the translation symmetry and hybridise the electronic states at the Dirac points [110]. The atomic sub lattice equivalency in graphene may also be broken; however, this does not affect translational symmetry. The gap is thought to have opened as a result of the graphene layer and SiC substrate contact breaking the K’ and K points symmetry in the zone (Brillouin). The EG emerged on the surface of a SiC substrate with a 6 6 domain pattern and a 6 3 6 3R30 periodicity atomic pattern [111]. The use of EG on SiC, which is similar to glassy carbon, carbon nanotubes and boron-doped diamond, has potential for developing an electroanalytical platform in a broad variety of biosensors. At physiological pH, EG-based biosensors can resolve all four nucleic acid bases and differentiate uric acid, ascorbic acid and dopamine despite the existence of edge and plane errors due to the electrochemical performances [112]. When SiC wafer prices remain exorbitant, EG can be readily incorporated into current electronic processes, and the bandgap provides an electrical off-state that is helpful in graphene sensors and transistors.

Gas backpressure of up to one bar of silane or, more often, argon may be provided to produce more uniform layers. With this method, greater temperatures and slower speeds result in more uniform development. In addition, carbon might be deposited ahead of time to provide more carbon [113,114]. However, despite the fact that optimization of these growth processes has resulted in relatively uniform monolayer graphene on SiC samples, a surprising amount of variance in electron transport properties still exists. In addition, epitaxial growth may result in the formation of undesirable polar faces such as Si-face or C-face, both of which detract from the quality of the final graphene product. Formation of graphene on the Si face is preferable because it leads to consistent graphene development. The amount of graphene layers, which varies with heating temperature, may be controlled using this approach with relative ease [115]. The production of graphene is controlled by the silicon polar effect. Furthermore, it was discovered that the silicon face of graphene has less orientation errors than the carbon face, making it the ideal face for the fabrication of high-quality epitaxial graphene. However, the expensive cost of silicon carbide and the necessity for high temperature (1000 °C) for this reaction mean that this method is unsuitable for the epitaxial graphene-based electronics sector. The atomically fat surface of hexagonal boron nitride makes it an ideal substrate for growing high-quality epitaxial graphene. Numerous studies have shown that the epitaxial graphene development on a hexagonal boron nitride substrate is characterised by a lack of impurities and a negligible quantum Hall effect [116,117]. As there is a lack of information on the growth processes and interaction between graphene and the substrate, this synthesis approach is still being evaluated at this time.

#### 2.2.3. Pyrolysis

Using plasma as a microwave discharge to create graphene with the necessary properties is one of the most promising methods currently being explored [119]. Using the pyrolysis process, the chemical synthesis of graphene through the bottom-up method may be accomplished using the solvo-thermal method. During the heat reaction, for example, sodium and ethanol were taken in a molar ratio of 1:1 in the vessel. Another example is the sonication-induced pyrolization of sodium ethoxide. This method may significantly improve the detachment of graphene sheets. As a result, the graphene sheets produced may be measured up to 10 m in length. Transmission electron microscopy, selected area electron diffraction and Raman spectroscopy are employed to investigate crystalline structure, various layers, band structure and graphitic nature [120]. Dato and co-workers [121] discovered a new way to synthesise high-quality graphene. Ethanol droplets were added to the plasma of argon that had been created in a quartz tube. Afterwards, the ethanol vaporised into smaller droplets, which were collected on nylon membrane filters. Graphene materials with two to four layers were produced and were readily dispersed by sonication. Transverse electron microscopy (TEM) and Raman spectroscopy were used to analyse graphene sheets, revealing high purity graphene production and the existence of single and multiple graphene sheets. Subrahmanyam and associates [122] used the arc discharge approach to produce graphene flakes. The arc discharge procedure was carried out in a stainless-steel chamber cooled by water, with the anode and cathode made of graphite and separated by only 2 mm. Graphene was extracted from deposits on the chamber’s inner walls since the cathode deposits were mostly MWCNT. High-current and high-pressure hydrogen were optimal for graphene production. A weight graphene yield of 10–20% was achieved relative to the anode weight under these circumstances. There are some similarities between this growth mechanism and the CNT synthesis process, with the main difference being that the lack of a catalyst and substrate makes the creation of 1D structures more difficult.

#### 2.2.4. Substrate-Free Gas-Phase (SFGP)

SFGP is a comparatively novel technique for synthesising graphene compounds without the requirement for substrates using a gas phase approach. At high pressures, an emulsion combination of fluid ethanol and Ar gas is transported to microwave-created plasma. Graphene is created by vaporising and dissociating ethanol droplets in the plasma zone for the duration of one second. This method is said to generate around 2 mg/min of graphene from 164 mg/min of ethanol input. Using this technique, Dato and Frenklach [123] examined the effects of several carbon precursors. Precursors such as isopropyl alcohol and dimethyl ether were discovered to produce graphene nanosheet. However, the result needed to be morphologically characterised and undesirable amorphous components removed [123]. The results also indicated that SFGP may have a process similar to that of soot production, allowing for the employment of less expensive precursors [123]. However, there are no thorough parametric studies for this technique, and further study is needed to determine the mechanism of SFGP. Overall, SFGP has a great potential for commercialization due to its ability to generate clean, high-quality graphene.

#### 2.2.5. Total Organic Synthesis

The unique characteristics of PAHs are used to produce graphene in complete organic synthesis. PAHs are sometimes described as two-dimensional graphene segments containing only sp^2^ carbons because of their intermediate structure between molecular and macromolecular phases [124]. Suitable aliphatic chains may also be easily substituted for PAHs to alter the product’s solubility [125]. The selection of appropriate precursors to produce high-yield and -quality graphene through a simple reaction pathway is the most important stage in this procedure. Total organic synthesis was used to construct 2D (graphene nanorods) GNR with lengths up to 12 nm [126]. The restricted size range of PAHs, however, may impact graphene quality by lowering graphene solubility and causing side reactions as a consequence of increased molecular weight [127]. It was shown by Yan et al. [128] that the resultant graphene possesses stable dispersibility despite its huge size, which solved these problems. Graphene manufacturing has advanced significantly due to complete organic synthesis, although side reactions and accurate parametric control have slowed commercialization.

#### 2.2.6. Template Route

**Figure 7:**
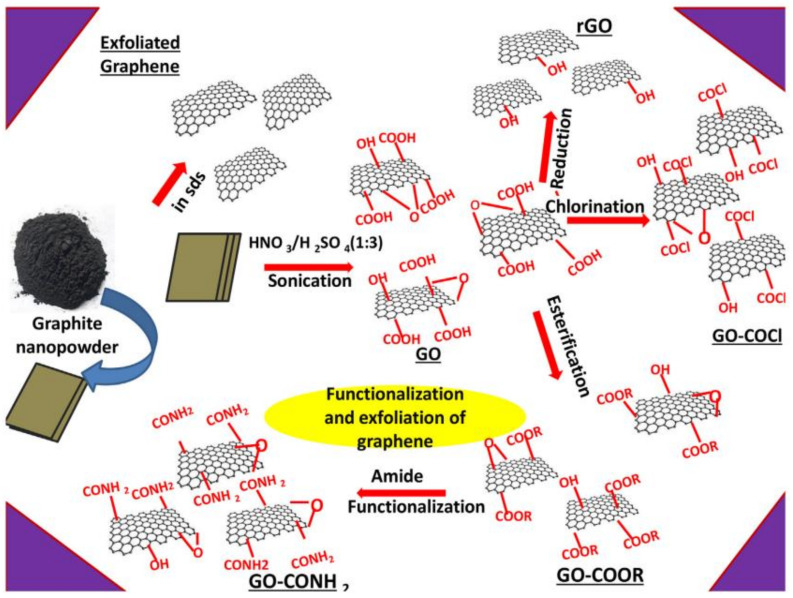
The schematic representation summarizing the chemical reactions performed for functionalization. (Reproduced from [132] with permission. Copyright 2015, Elsevier, Amsterdam, The Netherlands).

One-dimensional metal oxide, or synthetic polymer templates, has reportedly been used to manufacture graphene equivalents with superb quality [129]. Wei et al. [130] developed the template technique. When it comes to graphene, the first step was to produce a ZnS ribbon as a template using CH_4_ as a carbon source, followed by CVD template growth. The remaining ZnS nanobelt was etched using HCl, which is a hydrochloric acid. Another template approach for the production of graphene is the array of graphene on a template of silica with pyrrole moiety–containing surfactant [122]. In an inert environment, the pyrrole molecule was polymerized and carbonised into single nanosheets. With non-hazardous ingredients and strict control and high yield (measured in grams), this method produced high-quality, stable single-layer graphene with a 0.6 nm thickness [122]. To synthesise graphene on a silica substrate, a soft-hard template method [131] was recently devised. A sandwich-like structure of SiO_2_/CTAB/pyrene was created utilising CTAB as the soft template and SiO_2_ as the hard template employing pyrene as the carbon supply. Thermal treatment at 900 °C for two hours in inert circumstances produced the graphene sandwiched between the soft–hard templates. Due to the time-consuming washing phase and the risk of irreversibly damaging the generated graphene during template removal [130], this approach is less attractive in terms of commercialisation. The schematic representation summarizing the chemical reactions performed for functionalization is shown in Figure 7 [132].

It is costly and difficult to produce high-quality graphene, despite the fact that it is considered one of the “future materials” by some. Graphene cannot be turned off since it is such a good conductor of electricity. To make graphene, hazardous chemicals and high temperatures are necessary. Because of this, it has a tendency to be poisonous. A major drawback of graphene as a catalyst is that it is easily damaged when exposed to oxidative conditions. As a result, researchers have explored derivatives of graphene. In the next section, we discuss the graphene derivatives in detail.

## 3. Graphene Derivatives (GPDs)

**Figure 8:**
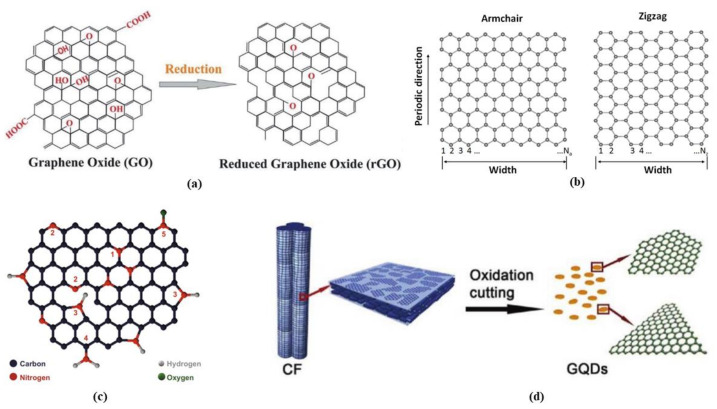
Structure of various graphene derivatives: (**a**) graphene and reduced graphene oxide (reproduced from [133] under common creative 4.0), (**b**) graphene nanoribbons (reproduced from [134] under common creative), (**c**) graphene nanowalls (reproduced from [135] under common creative 4.0), and (**d**) graphene quantum dots (reproduced from [136] with permission. Copyright 2012, American Chemical Society, Washington, WA, USA).

Simply described, graphene (GP) is a nearly atomically thick 2D sheet of hexagonal carbon rings. As a result, any material with these qualities may be classified as GP derivatives. As noted in the preceding section, several kinds of GPDs have been synthesised to date due to the requirement of decreasing manufacturing costs, boosting product yield, obtaining superior final product stability, and enhancing sensing capabilities. To understand how GPDs function as ECS modifiers, one must first understand their fundamental structural and chemical characteristics, as well as how these features relate to various production techniques. The structure of graphene derivatives is shown in Figure 8 [133,134,135,136].

### 3.1. Graphene Oxide

An oxygen-enriched form of GP has been named GO, as discussed in the previous section. Due to the presence of this negatively charged species, GO is much more water-soluble than GP in ECS and has a greater selectivity for binding positive or partial positive charge carrier species [137]. These oxygenation groups also cause problems in GO, such as the introduction of a few five- and six-membered rings. These imperfections obstruct ballistic electron transit and localise GO electrons, resulting in high sheet resistance and very poor conductivity [138,139]. In comparison to other GPDs, this renders it less suited as an electrode modifier. Cu, Fe, and new metal nanoparticles are often used to combat this issue [140,141].

### 3.2. Reduced Graphene Oxide

Through reduction, rGO may be generated from GO. Electrochemical reduction, chemical reduction, and thermal reduction may all be used to achieve this [142,143,144,145,146,147,148]. Each procedure has its own strengths and demerits. As a consequence, conductivity will be restored if the long-range conjugated network of GP can be repaired and oxygen groups can be removed. For ECS construction, rGO is a better WE modifier than either GO or GP because it possesses both the negatively charged groups of GO and the good conductivity of GP [149,150,151].

### 3.3. Graphene Nanoribbons

This is a cutting-edge GP material with a width of less than 100 nm. Simply described, GNRs are formed by cutting a single wall CNT or a multiwall CNT across their parallel axes and unfolding them to create a planar structure [152]. We can generate graphene oxide nanoribbons (GONRs) and decreased GONRs by using the same production procedure as GP (rGO NRs). GNRs with a width of less than 10 nm are now being researched [153]. Because of their higher surface-to-volume ratio and more exposed edge sites and electroactive defects, they might be useful electrode modifiers [154].

### 3.4. Graphene Nano-Walls

These are GP or GO sheets with sharp edges that have been constructed or grown in a parallel or vertical pattern. As a consequence, they have many sharp edges, which increases their specific surface area and hence increases the sensitivity of ECSs by offering more binding sites for target analytes [155,156].

### 3.5. Graphene Quantum Dots (GQDS)

The quantum confinement effect restricts electrical transmission in three spatial dimensions in GQDs, which are generally GP sheets with dimensions smaller than 20 nm. This may be accomplished by cutting GP sheets into little pieces or fractionalizing them. One of the most significant benefits of GQDs is that their edge sites may be adorned with different functional groups for improved electrochemical interaction with target analytes [157,158,159,160]. Other GDs, such as GP nanomesh (GPNM) [161] and graphene aerogel (GA), are also employed as electrode modifiers [162].

## 4. Defects in Graphene

Some early research looks at structural defects in carbon nanotubes [163,164] and graphite [165]. As a consequence, it is not hard to envision graphene becoming faulty at the atomic level as well. In reality, identifying the kinds of structural defects present in graphene precisely and quantitatively is challenging. Even for single-layer graphene that floats in the air, it is now feasible to view every atom in the graphene lattice [166]. Furthermore, the AFM (atomic force microscope) and SEM (scanning electron microscope) are extensively utilised experimental equipment for characterising nanomaterials [167,168]. As a result, theoretically anticipated configurations may be photographed directly. In general, defects in graphene may be divided into two groups: intrinsic defects, which are made up of non-sp^2^ orbital hybrid carbon atoms in graphene, and extrinsic defects, which are made up of non-sp^2^ orbital hybrid carbon atoms in graphene. The presence of non-hexagonal rings surrounded by hexagonal rings is typically the source of these flaws; extrinsic faults are the second kind. In graphene, non-carbon atoms disrupt the crystalline arrangement [169]. The structural reorganisation of carbon nanotubes in response to external energy shocks has been studied in earlier research on the migration of bulk crystal defects [170,171], and it is probable that faults are not always random and remain in one location. Instead, they move with a certain speed that depends on the activation barrier and temperature [172,173].

### 4.1. Intrinsic Defects

Stone–Wales defects, line defects, multiple vacancy defects, single vacancy defects, and carbon adatoms are the five types of intrinsic defects found in graphene. Defects in Stone–Wales: There are two neighbouring pairs of pentagonal and heptagonal rings in graphene that create the Stone–Wales flaws; this is due to a single pair of carbon atoms rotating around each other. No carbon atoms or dangling bonds are added or deleted as a result of the defects being developed. This defect’s production energy is estimated to be about 5 eV [174,175]. In high-temperature settings, Stone–Wales flaws may be deliberately created using electron radiation or fast cooling.

#### 4.1.1. Single Vacancy Defects

In graphene, one vacancy will be created if one carbon atom is absent from the ring of carbon that makes up the hexagon [176,177]. To reduce total energy, graphene suffers a Jahn–Teller distortion. Two of the three dangling bonds are linked to the missing atom and to each other. Due to geometrical constraints, one hanging link remains. The energy required to create the vacancy defect with such a dangling bond is greater than that required to form the Stone–Wales defect. The value of formation energy E_f_ 7.5 eV has been calculated [163,165].

#### 4.1.2. Multiple Vacancy Defects

The loss of another carbon atom following the loss of one will result in a two-fold vacancy defect. Instead of four hexagons, there are two pentagons and one octagon with no hanging connection. According to simulations, the production energy of this double vacancy defect may be as high as 8 eV [163]. Furthermore, due to its lower formation energy, the latter is more likely to be formed (about 7 eV). Furthermore, studies have shown that the likelihood of this defect is greater than that of the previous one. According to [177], the formation energy of the defect is between that of the first and second vacancy defects. The development of the second vacancy defect into more complex vacancy defects by rotating another bond would be a step further. Larger and more complicated defect restorations may arise from the removal of additional carbon atoms.

#### 4.1.3. Line Defects

Graphene starts to develop at various locations on the metal surface during the process of preparing it via chemical vapour deposition (CVD) [178]. Graphene polycrystallinity is virtually inevitable when using the CVD technique. Because of the unpredictability of growth, various crystallographic orientations appear in different places. Cross-fusion occurs when the graphene reaches a particular size [179]. Heptagons, hexagons, and pentagons provide the primary structural link between the two crystals. Despite the fact that the grain boundary is not exactly straight, the faults along it are not uniformly distributed. Another analysis has also revealed similar graphene line defects [180,181,182].

#### 4.1.4. Out-of-Plane Carbon Adatoms

Because of single and multiple vacancy defects, it is possible that the carbon atoms missing from the graphene plane will not be completely removed from the material. Rather, these carbon atoms travel to the graphene’s surface after separating from the original carbon hexagon ring. When the carbon atoms travel to a new in-plane point, a new bond is formed. New flaws may be created if carbon atoms contact with a flawless graphene sheet. Such flaws will cause the original planar structure to be destroyed, resulting in a three-dimensional structure. The graphene layer and the carbon adatom form a bridge. An atom of carbon travelling across the lattice produces this metastable dumbbell form. Two carbon adatoms in motion cause an inverse Stone–Wales defect. Carbon atoms that have migrated out of plane move swiftly and have a high energy of formation. As a result, different microscopic methods such as TEM, STM, and others struggle to capture them. Carbon adatoms outside of the plane have not been studied in depth. According to prior study on the activation process of activated carbon, carbon and oxygen atoms may travel along the carbon layer’s surface [183]. As a result, the presence of carbon adatoms that are out of plane may be confirmed. This flaw should, in reality, come in a number of spatial configurations. Furthermore, when the number of carbon atoms added rises, the structure becomes more complex [184]. The out-of-plane carbon adatom obliterates graphene’s two-dimensional crystal structure. Some flaws, in particular, alter the hybridization of the carbon atoms in the layer. Sp^3^-hybridization may be seen locally to some extent. A feasibility study including such flaws is now underway [185,186]. Clearly, making such flaws manageable is a difficult task.

### 4.2. Extrinsic Defects

Foreign adatoms and substitutional impurities are the two types of defects introduced into graphene. These two kinds of defects are discussed below.

#### 4.2.1. Foreign Adatoms

The CVD or strong oxidation procedures are always used to coat the graphene surface with metal atoms or oxygen-containing functional groups. Covalent bonding or weak van der Waals contact binds these adatoms to the closest carbon atoms. Foreign adatoms are the name for these types of flaws. Recent studies have shown that metal adatoms significantly affect graphene’s surface by causing migration [187]. Many theoretical studies of graphene flaws based on experimentation have been conducted. Adsorption and surface motion [188] and the relationship between magnetic, electrical, and defect features [189] were some of the topics addressed in this research. The most prevalent types of foreign adatoms are oxygen atoms and oxygen-containing functional groups, such as carboxyl or hydroxyl groups. The Hummers technique, a kind of graphene synthesis process, is to blame for this flaw. The research of Hummers for the preparation of oxidised graphite led to the development of this technique. The fundamental technique for graphene has been refined by numerous researchers [190], but the basic process remains the same. Throughout the procedure, strong oxidants such as potassium permanganate, nitric acid and concentrated sulfuric acid are employed. The graphite sheet is exfoliated and oxidised using a strong oxidising agent and then reduced using a thermal or chemical reduction process [191,192]. To remove oxygen-containing functional groups, a reduction agent is employed. Finally, this technique was used to make graphene [193]. During the following reduction process, the oxygen atoms in graphene are difficult to fully eliminate. Thermal reduction or a reducing agent will never completely remove all of the oxygen atoms from the final graphene product. Photoelectron spectroscopy may also be used to determine the oxygen concentration and existing form [194].

#### 4.2.2. Substitutional Impurities

Carbon atoms may be replaced in graphene by other elements with three chemical bonds, such as boron and nitrogen. These heteroatoms are the building blocks of graphene substitutional impurity defects. In graphene, boron and nitrogen atoms may exist separately. They may also exist at the same time due to method control [195]. By controlling the process, boron and nitrogen atoms are intentionally inserted into the graphene. The explanation for this is that graphene that has been doped with nitrogen, or boron has high conductivity and catalytic activity [196]. Its conductivity and other characteristics, however, are outstanding. The electron cloud surrounding graphene in the immediate region alters when nitrogen and boron are introduced. Furthermore, this increases the activity of these areas [197]. Similar to substitutional impurities, boron and nitrogen atoms have their own distinct features that affect graphene’s properties.

### 4.3. Double Graphene Structure Defects

**Figure 9:**
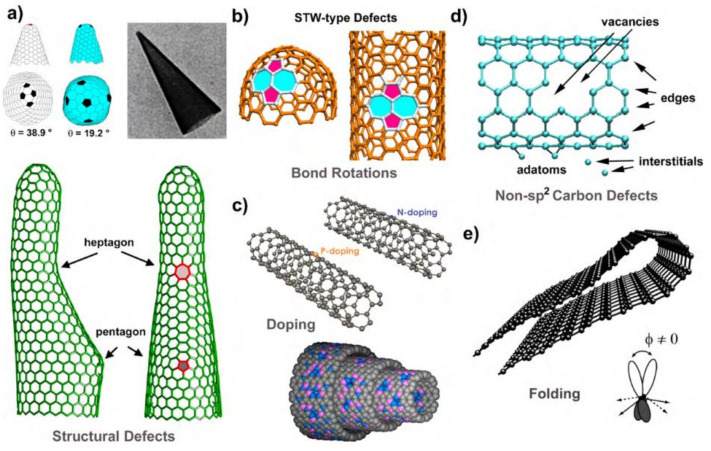
Representations of defects in graphene-like materials using pictorial models. (**a**) defects in the hexagonal sp^2^ hybridised carbon lattice, (**b**) Stone–Thrower–Wales defects, (**c**) replacement of carbon with another element inside the hexagonal lattice, (**d**) carbon defects, such as vacancies, edges, adatoms, interstitials, carbon chains, etc., that are not sp^2^ hybridised, and (**e**) consequence of considerable deformation of graphene, folding-induced defects. (Reproduced from [199] with permission. Copyright 2010, Elsevier, Amsterdam, The Netherlands).

In some cases, there exists a special type of defect in the graphene structure that is different from both intrinsic and extrinsic defects, it and is known as the double graphene structure defect. The graphene creates a graphite-like structure when it is built in layers. A defect-free graphene sheet will have no chemically bonded carbon atoms. There are intrinsic flaws in graphene such as dangling bonds, holey sheets, and carbon atoms in the migratory state that will form new chemical connections even with only two layers of graphene bonded together [198]. The structural flaws will be more complicated if the stacking procedure includes additional layers of graphene. These intricate flaws are therefore likely to have an impact on the construction material’s macrostructure. It will also have an impact on the material’s physical and chemical characteristics. Because graphene nanosheets and monolithic graphene are not infinitely big in space, the creation of graphite structures in distinct stacking areas must include concurrent domain operations. If the domain processes are not up to par, the material will be devoid of long-range order. This will result in material flaws as well. Figure 9 shows a variety of graphene-like materials with various defects [199]. Figure 9a shows the defects in the hexagonal sp^2^ hybridised carbon lattice that creates major structural changes due to the existence of pentagon or heptagons. Figure 9b shows no major structural changes that are caused by topological defects, also known as Stone–Thrower–Wales defects. Carbon–carbon bonds are rotated 90 degrees to generate 5-7-7-5 pairs, as seen above. Figure 9c shows the replacement of carbon with another element inside the hexagonal lattice (here, N and P) or random doping of CNTs with B and N. Figure 9d shows the carbon defects, such as vacancies, edges, adatoms, interstitials, carbon chains, etc., that are not sp^2^ hybridised. Figure 9e shows that as a consequence of considerable deformation of graphene, folding-induced defects are formed. When the orbital axis vector is known, it is referred to as the orbital axis vector (POAV). The degree of “pyramidalization” and the degree of hybridization are shown by the angle between the POAV and a direction (i.e., a bond). The orbitals are in a pure pz orbital for =90 (planar system) and are hybridised in sp^2^.

## 5. Properties of Graphene Materials

As previously discussed, graphene and its derivatives are known for their improved properties. In this section, we discuss these properties in detail.

### 5.1. Mechanical Properties

**Figure 10:**
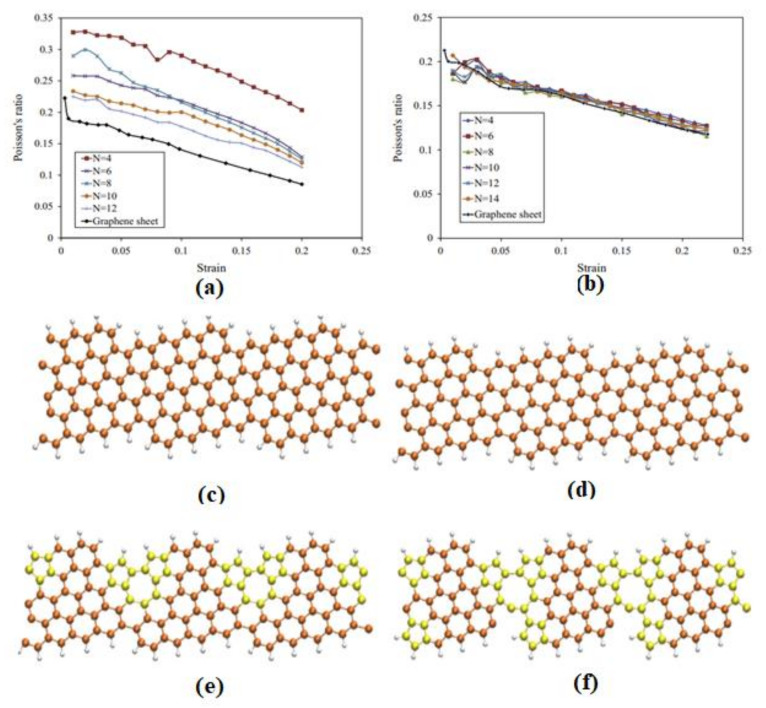
The Poisson’s ratio of graphene nanoribbons with different widths. (**a**) Armchair nanoribbons and (**b**) zigzag nanoribbons. A graphene nanoribbon with 10:890 chirality under uniaxial tensile loading: (**c**) zero strain, (**d**) 10% strain, (**e**) 12% strain, and (**f**) 16% strain. (Reproduced from [207] with permission. Copyright 2014, Elsevier, Amsterdam, The Netherlands).

Graphene’s exceptional mechanical qualities are one of the reasons it stands out as a material and as a reinforcing element in composites. Graphene’s extraordinary mechanical qualities come from the stability of the sp^2^ bonds that make up the hexagonal lattice and fight against several types of in-plane deformations. Graphene’s planar density is only 0.77 mg/m^2^, making it one of the lightest materials known. As far as we know, its crystal structure is likewise the strongest and hardest of any substance out there. Its elastic modulus is 1.1 TPa, whereas the elastic modulus of standard steel is 200 GPa; hence, its tensile strength is much higher. Graphene is 100 times stronger mechanically than steel, with a breaking strength of 42 N/m. The mechanical characteristics of free-standing monolayer graphene were initially determined by Cao and co-workers [200] using nanoindentation in an AFM, and the results established graphene as “the strongest material ever measured”. Intriguing mechanical properties of graphene materials, with potential nanoelectromechanical applications, are on the horizon. Li et al. [201] conducted the first thorough experimental analysis of the elastic characteristics and strength displayed by pure graphene. Studies show that graphene exhibits cracking and quasi elastic behaviour. Min et al. [202] used molecular simulations to calculate the fracture strain, shear modulus and shear strength of graphene as a function of chirality and temperature. Fracture stress was determined to be 97.54 GPa, while shear strength was found to be 60 GPa when the graphene sheet was very flat. The mechanical characteristics of graphene have been published by Ovid’ko et al. [203], who found that pure graphene has a Young’s modulus of 1 TPa and an inherent strength of 130 GPa, which fits the computer models they used to compare. The Young’s modulus of nanometre-thick graphene sheets is 0.5 TPa, according to Frank et al. [204]. Despite the fact that the literature shows graphene’s mechanical characteristics to be inconsistent, it is clear that graphene has promising mechanical properties and is a worthwhile material for future study and use in the composites field. Zigzag fractures may occur near the stress limit due to graphene’s inherent defects, including vacancies, dislocations and grain beads. As a result, it is critical to better comprehend these flaws and to propose fresh research ideas that will have a good influence on graphene’s uses [205]. Suk et al. [206] used an atomic force microscope (AFM) in contact mode to undertake a finite element method (FEM) study on predicting mechanical characteristics of GO. As a novel tool for determining mechanics, AFM measurement paired with FEM was used to evaluate the elastic modulus of thin GO ultra-thin films. According to this recent research, GO ultra-thin films have a Young’s modulus of 207.6 ± 23.4 GPa and a thickness of 0.7 nm, which is regarded as equal to pristine graphene. The graphene oxide sheets’ pre-stress was found to be 39.7–76.8 MPa, which is one order of magnitude less than that of mechanically exfoliated graphene. The elastic modulus and pre-stress of thin graphene oxide sheets may be determined using this unique hybrid technology (combining AFM and FEM mapping). Table 1 summarises the most current studies on graphene derivatives’ mechanical characteristics. Figure 10 illustrates the Poisson’s ratio and graphene nanoribbon strain percentage [207].

### 5.2. Electrical Properties

**Figure 11:**
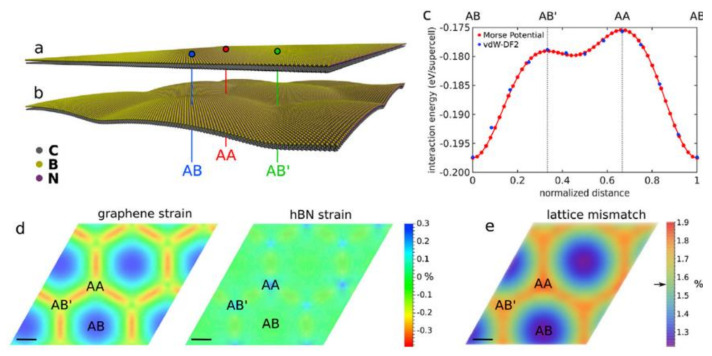
The electrical characteristics of a graphene/hexagonal boron nitride bilayer. (**a**) layer of graphene/h−BN bilayer before stress, (**b**) graphene/h−BN bilayer structural model after full relaxation, (**c**) the interlayer binding energy curve for each super monomer, (**d**) strain maps for graphene and hexagonal boron nitride, and (**e**) lattice mismatch. (Reproduced from [246] with permission. Copyright 2017, Elsevier, Amsterdam, The Netherlands).

Graphene is one of the greatest electrical conductors on Earth, which is why it has garnered so much interest from scientists developing molecular electronics. Graphene’s electrons are able to move at extraordinarily high velocities because to the special arrangement of its carbon atoms, allowing them to avoid the considerable scattering that wastes energy in conventional conductors. Because the electrons in graphene do not appear to slow down or localise, scientists have determined that it can conduct electricity even at the limit of supposedly zero carrier concentration. New, massless quasiparticles are created as electrons circling carbon atoms react with the periodic potential of graphene’s honeycomb structure (so-called massless Dirac fermions). Graphene retains its electrical conductivity indefinitely. Graphene and polymer composites have been the subject of much research aimed at increasing or introducing electrical conductivity (Table 2). There have been a number of notable efforts made by other researchers to achieve this aim, as well as challenges. Graphene materials have been added in different weight and volume percent amounts to a variety of polymer matrices ranging from thermoplastic to thermoset, with encouraging results in each case [236,237,238,239,240,241,242,243]. Low-temperature methods such as melt blending, solution casting, in situ processing, and chemical vapour deposition may be used to assess electrical conductivity. Adding graphene elements to a secondary polymer or filler may improve electrical conductivity [244]. Dispersion in a polymer matrix of graphene particles is problematic because of the creation of agglomerates, and the addition of graphene is limited to a set amount. As a consequence, the conductivity is low. Most previous research has focused on simple composite blends, and most researchers did not explore complicated blends or hybridization as a way to move beyond the constraint of adding graphene material above a particular level. This would have enabled the addition or usage of other potential materials to enhance mechanical and electrical qualities alongside graphene. The stability, i.e., thermal and mechanical stability of the composites have significantly increased, and the adaptability of graphene-reinforced polymer nanocomposites suggests that they might be used in flexible devices, sensors and packaging [245]. Furthermore, graphene reinforcement combined with a secondary polymer or filler may increase electrical conductivity [244]. The electrical characteristics of a graphene/hexagonal boron nitride bilayer are shown in Figure 11. Figure 11b shows a graphene/h-BN bilayer structural model after full relaxation. It is easy to see that the relaxation model’s vertical sample plane is twisted. The interlayer binding energy curve for each super monomer is shown in Figure 11c. Strain maps are shown in Figure 11d for graphene (left) and hexagonal boron nitride (right). Figure 11e demonstrate lattice mismatch. Table 2 summarises the most current studies on graphene materials’ electrical characteristics.

### 5.3. Thermal Properties

**Figure 12:**
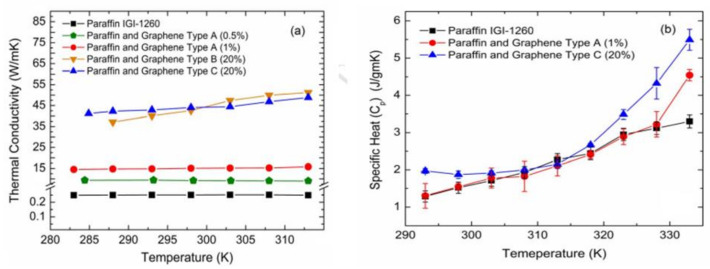
Hybrid graphene–PCM thermal characteristics: (**a**) temperature-dependent enhancement factor of the thermal conductivity of graphene—paraffin composites with various graphene loadings. For comparison, the outcomes for pristine paraffin are also shown: (**b**) temperature-dependent specific heat of the composites and the reference pure paraffin. (Reproduced from [262] with permission. Copyright 2013, Elsevier, Amsterdam, The Netherlands).

The thermal qualities of a material establish its features, but its conductivity is determined by its atomic structure. When materials are organised on a nanometre scale, their thermal characteristics vary. Theoretical and experimental research has demonstrated that graphene crystals have infinitely high inherent heat conductivity. Thermal conductivity (κ) of a material is inversely proportional to temperature gradient (Q″ = −κ ∇T) and inversely proportional to heat flow per unit area (Q″) (e.g., in W/m^2^). In this relationship, heat is shown to flow negatively from high to low temperatures. According to a mathematical formula that uses λ and v as the mean free path and phonon group velocity, the thermal conductivity immediately correlates to the specific heat by κ ≈ ∑Cvλ [260]. Graphene has an in-plane thermal conductivity at ambient temperature that is around 2000–4000 W m^−1^ K^−1^, making it one of the highest of any known material. Isotopically pure samples (0.01% 13 C instead of 1.1% natural abundance) with big grains reach the higher end of this range, whereas isotopically mixed samples or those with smaller grain sizes reach the lower end. These values will naturally decrease if more phonon scattering is introduced owing to more disorder or even residue during sample preparation. Graphene’s in-plane thermal conductivity reduces significantly when this 2D material comes in contact with a substrate or is confined in graphene nanoribbons, despite its high room-temperature value for freely suspended samples (GNRs). Given that phonon propagation in an atomically thin graphene sheet is anticipated to be particularly sensitive to surface or edge disturbances, this behaviour should come as no surprise. At ambient temperature, the thermal conductivity of graphene over SiO_2_ (600 W m^−1^ K^−1^), graphene encased in SiO_2_ (160 W m^−1^ K^−1^), and supported GNRs (80 W m^−1^ K^−1^ for 20-nm-wide samples) was measured and approximated, respectively. Coupling and dispersion of graphene phonons with substrate vibrational modes causes a drop in heat conductivity for SiO_2_-supported graphene [261]. Table 3 lists the thermal conductivity values of several graphene compounds. Thermodynamic properties of hybrid graphene–PCM [262] are shown in Figure 12.

### 5.4. Non-Toxic Nature

Since the beginning of research on graphene materials, toxicity and safety have been a focus. Graphene, however, has a number of characteristics that make it potentially suitable for biomedical applications. According to the expanding studies of graphene-based materials (GBMs) in biomedical applications, the hydrophobic forms of GBMs that collect on cell membrane surfaces are more dangerous compared to the most hydrophilic forms of GBMs that penetrate the cellular membrane [274,275,276,277,278,279]. For the same kind of GBMs, the influence of particle size on cell feasibility has yet to be investigated [280].

**Figure 13:**
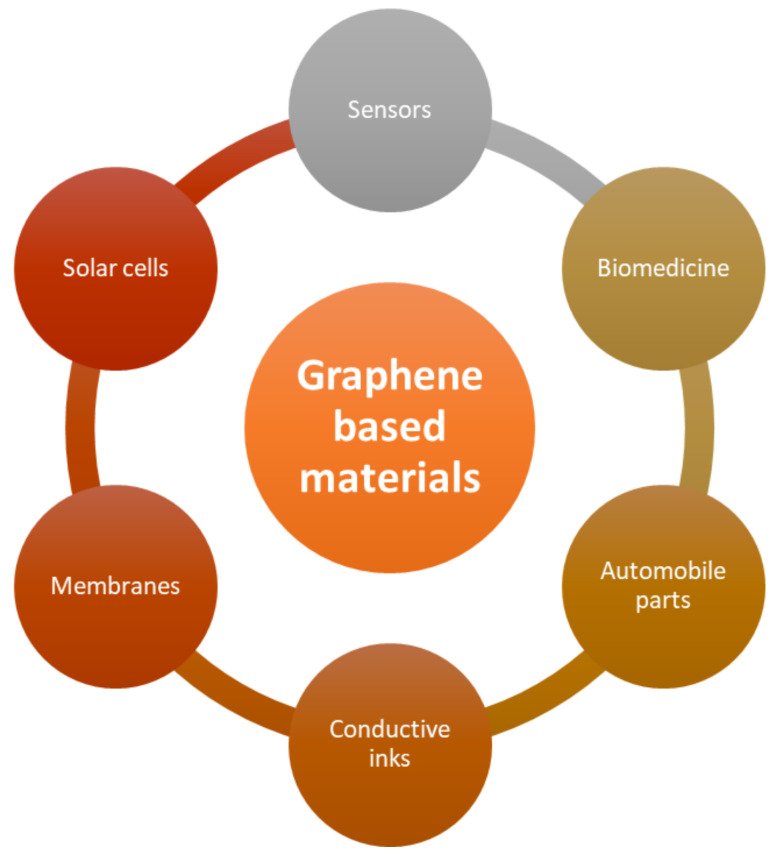
Graphene materials’ promising application areas.

Due to these properties, graphene and its derivatives find various applications [281]. By strengthening polymer structures with graphene, graphene composites have found a wide range of applications. Graphene-based polymer nanocomposites have three primary properties that characterise their performance: Nanoscale inorganic ingredients and their fluctuation in characteristics, as documented by many studies of their major change according to their size, and nanoparticle configuration and development of huge polymer/particle interfacial area. Figure 13 depicts a distinct application/research area for graphene-based composites.

## 6. Biosensors for Sweat Analysis

### 6.1. Sweat Chemistry

Understanding sweat composition, biomarker physiological levels, and blood constituent correlations is critical for developing smart health monitoring devices. Sweat contains indicators that may represent an individual’s overall biomolecular condition as well as their fitness level. Perspiration is primarily a means of thermoregulation in the body, and metabolites, biomolecules, ions, hormones, amino acids, proteins, and peptides are all expelled in sweat during this process. The primary physiologically significant indicators and their dynamic concentration levels in human sweat are listed in Table 4. Sweat contains substantial concentrations of lactate [282,283,284,285], glucose [286,287,288,289,290], uric acid [291,292], ascorbic acid [293,294,295], cortisol [296,297], tyrosine [298,299], ethyl glucuronide [300], F17464 [301], Na^+^ [302,303,304,305], Cl^−^ [306,307], K^+^ [308,309], pH [310,311,312], NH^4+^ [313], Ca^2+^ [314], Zn^2+^ [315] and Cd^2+^ [316]. Because of their high sensitivity and ease of development in small electronic circuits, the majority of sweat sensors use amperometric and potentiometric transduction methods. For different sweat sensing applications, sensitive voltammetric approaches such as SWASV (square wave anodic stripping voltammetry) and DPV (differential pulse voltammetry) have recently been used [291]. The secretion mechanisms and analyte partition methods in the sweat fluid are linked to the amount of sweat components [317]. The majority of perspiration is produced by the eccrine sweat gland, a kind of exocrine gland that generates sweat first and then transports it to the epithelium surface through a dermal duct. The potassium–sodium pump transports Cl^−^ and Na^+^ ions between the secretory coil and blood serum, creating a hypotonic osmotic pressure gradient that pulls fluid into the eccrine glands. As the sweat travels through the dermal channels, Na^+^ and Cl^−^ are reabsorbed. High Na^+^ levels may be caused by an increase in sweating rate that is greater than the rate of reabsorption, which occurs when the rate of sweating exceeds the rate of reabsorption. Sweat electrolyte concentration monitoring gives important information about the human body’s chemical and physical status. It is possible to detect electrolyte loss during ultra-endurance exercise by monitoring the quantity of sodium in the sweat, since sodium is a fundamental component of perspiration. Excess sodium in sweat is linked to hyponatremia and low water content in the blood (dehydration). Human health is threatened, and physical and mental health may suffer as a result. Cystic fibrosis, a progressive genetic disease caused by mutations in the transmembrane conductance regulator channel, primarily affects the lungs and other internal organs, and the unequal distribution of sodium in sweat is clinically relevant in its diagnosis. Cystic fibrosis progress may be monitored non-invasively using sweat-based biomedical technologies. For accurate diagnosis, the exact concentration of Cl^−^ or Na^+^ is generally established. The inability of eccrine glands to reabsorb Cl^−^ results in lung destruction and a rise in the Cl^−^ concentration in sweat [318]. The iontophoretic examination of sweat offers quick screening and diagnosis of cystic fibrosis, particularly in infants. The partitioning mechanisms of biomarkers from blood or ISF to sweat fluid are poorly understood. In order to maintain dynamic concentration levels of analytes, charge, size, and sweating rate play a critical role. Energy is required for the transport and reabsorption of hypo-osmotic Na^+^ between cell membranes and the duct wall [319]. Blood glucose oxidative phosphorylation is a major source of energy for sweat gland function. As a result, exogenous glucose serves as a source of energy for sweating [320]. The eccrine gland’s energy metabolism produces lactate as well when O_2_ is scarce [321]. When there is a requirement for a large quantity of energy in a short amount of time, glucose is converted to lactate (anaerobic glycolysis). Long-term high-intensity exercise depletes aerobic glycogen stores; thus, anaerobic metabolism steps in to provide the energy and lactate needed. In lactic acidosis, the blood lactate levels are elevated. As a result, a high quantity of sweat lactate suggests a low degree of tissue oxygenation, particularly in muscle cells. Because low oxygen levels are linked to weariness, muscular weakness, and muscle cramps, non-invasive lactate monitoring is critical for persons who engage in high-intensity activities. Lactate also reflects a poor oxidative metabolism and hence offers information on the extent of restricted blood flow (pressure ischemia) [322]. Pressure ischemia may develop to more serious clinical problems, such as decubitus ulcers [323], which are open skin wounds produced by body weight pressing the skin against a hard surface.

Hypokalaemia and metabolic alkalosis are linked to chronic renal disease [324]. Persistent hypokalaemia causes peripheral neuropathy, muscle disease, and an increased risk of falling and paralysis [325]. Hyperkalaemia (excess potassium in the blood) causes decreased urine output, cardiac arrest, irritability, nausea, and loss of gastrointestinal tone [326]. As a result, measuring the potassium content in sweat reveals the human body’s fundamental physiology. Ammonium is a by-product of protein degradation, and its excessive concentration in blood plasma suggests metabolic problems, nutritional issues, and liver dysfunction [313]. Ammonia is converted to urea in the liver and excreted via urine. The dispersed ammonia in sweat is ionised at high pH and is entrapped in the secretory cell wall, resulting in a higher quantity of ammonia in perspiration (millimole range) than typical blood serum concentrations (11–32 mmol/L). As a result, elevated sweat ammonia levels might be a signal for those with hepatic illnesses such as cirrhosis or hepatitis. Furthermore, chemicals such as heavy metals, alcohols, and narcotics that are excreted during the body’s toxin management process must be monitored. Sweat is generated in a variety of ways, including physical activity, mental stress, heat, and chemical stimulation, and the sweat composition varies depending on the sweat extraction method. As a result, for a promising sweat profiling application, an appropriate sweat stimulation process must be used. During strenuous physical activities, the dynamic biomarker level offers information on the wearer’s physiological and fitness status, as well as the degrees of metabolic activity. However, medical analysis requires an equilibrium level of sweat ingredients, which may be achieved by promoting sweat production locally using the iontophoresis procedure [327].

### 6.2. Features of a Good Biosensor

**Figure 14:**
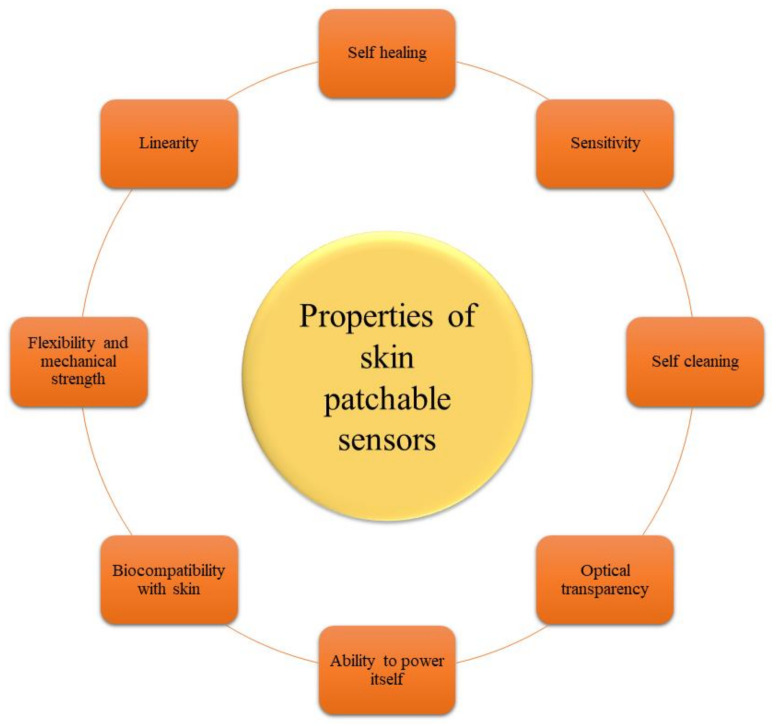
Skin-patchable sensors have a number of desirable characteristics.

Some of the necessary characteristics for a viable skin-patchable sensor and its parts are listed in Figure 14. Linearity, biocompatibility, sensitivity, transparency, flexibility and mechanical strength, self-powering ability and self-healing are some of these characteristics [328]. For a successful attachment of a device to the human body, biocompatibility, flexibility and high mechanical strength are required, and these characteristics are discussed.

#### 6.2.1. Substitutional Impurities

Because patchable skin sensors are subjected to very high stresses, measurement linearity is critical. The calibration procedure is complicated by linearity deviation, which is a significant constraint in most resistive-type sensors. When the sensors are stretched, nonlinearity occurs, which is caused by the change of the nanostructures from uniformity to non-unformal topology [329].

#### 6.2.2. Sensitivity

Sensitivity is classified as the relationship between variation in electrical signal (capacitance and resistance) and stress or strain. Skin-patchable sensors benefit from stretchable conductors with a high peizo-resistance. When micro- and nanostructures break down and link with one other, this process is what causes sensors to be sensitive to little changes [330]. Tunneling peizo-resistance and high-pressure sensitivities are provided by microstructures with fractured or cracked microstructures [331]. A range of processes and designs, when combined, may result in an increase in sensitivity.

#### 6.2.3. Flexibility and Mechanical Strength

One of the most important considerations while making a skin-patchable electrode is to achieve feasible contact between the sensor and skin with the least amount of invasiveness and contact resistance possible [330,332]. This necessitates a greater focus on the design of component materials that are both strong and flexible. With elastic modulus ranging from 10 to a few hundred kPa, normal human skin may deform up to 15% [333]. As a result, patchable skin sensors should have enough stretch ability to stay connected to the skin and adjust to mechanical bending and stretching during movement. Because flexibility is related to the third power of material thickness, it is essential to change the flexural strength of the sensor’s component materials during fabrication [334,335,336]. There are many studies available on the fabrication of devices with inorganic or organic components on extremely tiny substrates, which may result in micrometre-sized bending radii even when employing materials with very high elastic moduli [337,338]. The utilisation of high-fracture-resistance materials such as carbon nanotubes, graphene [339], metal oxides, polymers and hydrogels may be a more operative way to create reflexively robust electronics. Aside from integrating effective substance and decreasing thickness, the device’s morphological and structural design also contributes to its mechanical stability [340]. Soft lithography has great promise in this regard, since it provides soft moulds for imprinting specific materials, enabling the creation of complex 3D morphologies. It also allows for the cost-effective use of elastomeric substance for the integration of nanoscale components into planar and nonplanar topographic surfaces [341]. Another popular method is to create an island-bridge architecture, in which conductive bridges are connected to the active part, which are referred to as islands [342,343,344,345]. These interconnects are designed to allow for overall device stretching while reducing strain on specific functional components. As a consequence, these interconnects must be able to resist the repeated stresses that the human body experiences on a regular basis. Matsuhisa et al. [346] described a printable elastic conductor comprising AgNPs, which were created in situ by combining Ag flakes in nano-range, a surfactant and fluorine rubbers. The surfactant, heating method, and elastomer molecular weight all had an impact on AgNP production. At 0 percent strain, the printed elastic composite had a conductivity of almost 4000 S cm^−1^ and 935 S cm^−1^ when stretched up to 400 percent. Another method for creating skin-patchable devices with greater scalability is additive printing (inkjet and 3D printing) [347,348,349,350,351,352,353]. Polymers, biomaterials, semiconductors, ceramics and metal nanoparticles are among the materials that may now be used [354,355,356]. In addition, by interacting with the target skin area, hybrid combinations of these materials may create functional devices [357,358].

#### 6.2.4. Self-Healing Ability

Self-healing functions are essential since devices can damage during normal operation [359]. Different components may self-heal and modify their function in the gadget using self-healing technology [360,361]. Self-healing substances show high resistance towards damage or tiny fractures, preventing them from spreading and increasing gadget robustness. Many materials are utilised as components in stretchy and wearable devices such as electronic skins and self-healing conductors [362,363,364]. Polymers utilised to construct devices with self-healing properties, however, tend to be viscoelastic and have low mechanical strength. Kang et al. [359] used rationally designed multistrength hydrogen-bonding interactions to overcome this limitation. Thus, a polymer film with a remarkable self-healing characteristic and mechanical stretchability was created using this supramolecular network. During the design of an electronic system, one of the major problems is the integration of various self-healing components. This problem was addressed by Son et al. [365], who studied nanostructures rebuilding during their interaction with a self-healing polymer (cross-linked) network. The polymer’s self-bonding property allowed various devices to be combined into a single electronic system. In a separate research study, Liu et al. [360] described wearable hydrogels with self-adhesive and self-healing characteristics that may convert mechanical stimuli of epidermal skin tissue deformation into readable electrical signals.

#### 6.2.5. Self-Cleaning Ability

The capacity of skin-patchable electrode sensors to self-clean ensures optimum operation and stability. Kar et al. [366] recently developed a self-sanitizing electronic skin that can imitate the ability of pressure-sensing real skin of humans. It was discovered [367] that nanoparticles based on carbon impart a superhydrophobicity surface of a sensor with an angle of contact at 150° and an angle of sliding at 10°. The superhydrophobic characteristic of the surface allowed water droplets, as well as dust particles and pollutants, to roll away [368].

#### 6.2.6. Optical Transparency

Wearable sensors should be translucent, so that they cannot be seen when applied on the neck and face, for ease and comfort [369]. Lan et al. [369] developed thermotherapy pads that are optically transparent and made of Ag nanowires on a PVA matrix. This film offers a 93.1 percent optical transparency and outstanding flexibility, as well as controlled heating and a quick thermal response. Chun et al. [370] developed a tiny and lightweight transparent pressure sensor made of graphene.

#### 6.2.7. Ability to Power Itself

Several methods have been discovered to integrate energy-producing and storing devices with wearable sensors [371,372]. Because skin-patchable devices need energy autonomy, they may be engineered to draw voltage from either the body of a human or the surrounding environment [373,374]. The mechanical movement of the human body may be used to extract power, which can then be transformed to electrical energy [375,376,377]. Human sweat, as in wearable biofuel cells [378], solar energy [379], the energy in the RF (radio frequency) spectrum may all be used to generate power [373,374]. TENG (triboelectric nanogenerator) is the most recent power-generation technology, which was originally disclosed in 2012 [380]. This operates on the tribo-electrification concept, which involves the creation of static opposing charges between two distinct materials that are placed face to face [381,382,383].

#### 6.2.8. Interfacing and Biocompatibility with Skin

Biocompatibility is an essential aspect in ensuring that the sensor is properly integrated with the skin and does not induce allergies on human skin, such as rashes and etching. There are three methods for sensors embedded on skin, each based on a distinct way of fixing the sensor to the skin, such as hard-soft integration [384], epidermal or tattoo-like integration [375], and functional substrates [385]. The elastic modulus of the materials used as temporary epidermal tattoos is comparable to that of the skin, allowing for contact and adherence between the skin and the sensor [386]. In most sensors based on epidermal tattoo, silicone polymers such as Ecoflex, PDMS, and Solaris played the role of substrates. Polymers such as polyimide, polyester, PET (polyethylene terapathalate) and PVA (poly(vinyl alcohol)) have also been utilised as substrates that may be merged with the body of a human at various places in addition to silicone materials [384]. Skin-mountable integrated circuits may be built using this technique. The third functional substrates approach includes combining several functional substrates and thin films to fabricate sensors for a specific application [387]. As a result, all of these variables must be considered when selecting a suitable material for skin-patchable electrodes. The contact between the sensor and the skin is the other most essential element. The important feature at the interface is the sensor’s improved adherence to the skin, allowing it to actively evaluate strain, perspiration, blood pressure, and other factors. Biocompatibility is also a significant consideration.

### 6.3. Graphene and Its Derivatives for Biosensors

Nanomaterials based on electro-chemical sensors have gained a lot of interest as nanotechnology has developed. Among these, graphene’s exceptional properties—including its great mechanical strength, enormous surface area, superior electrical and thermal conductivity, high thermal stability, and strong biocompatibility—have led to its widespread use. Nanomaterial graphene is a single-atom-thick sheet of special carbon atoms called Sp^2^ hybrids that is organised in a honeycomb lattice. Graphene’s vast surface area and porous structure, made possible by its unusual electrical structure, make it an excellent alternative for versatile engineering. The reaction time and detection sensitivity of biosensors are both enhanced by graphene’s exceptional electrochemical characteristics, such as its rapid electron transfer rate, low charge transfer resistance, and good electrical and thermal conductivity. The graphene-based electrochemical biosensors have been used for a wide variety of applications because they take advantage of graphene’s structural and electrochemical properties. Some of these include the detection of glucose, cholesterol, ascorbic acid, uric acid, and cancer biomarkers. The graphene-based sensors have a shorter reaction time, lower price tag, and smaller footprint than their predecessors. These graphene-based sensors provide a number of benefits for sensing, such as the ones listed below.

(1) Splendidly large surface area: For single-layer graphene, the predicted surface area is 2630 m^2^/g, leading to a high density of bound recognition components or analyte molecules. This aids in the downsizing and high detection sensitivity of the apparatus.
(2) Exceptional electrical characteristics and electron transport abilities: Graphene’s sp^2^-hybridized carbon atoms create a massive free-flowing electron conjugate system. Graphene is an appealing material for electrochemical sensing because of its unique characteristics.
(3) Extreme mechanical toughness and malleability: Graphene, a two-dimensional material with a thickness of only 0.335 nm, has a hardness greater than diamond because of its strong C=C bonding in the atomic plane. However, unlike diamond, the interlayer bonding of graphene is weak owing to the action of Van der Waals forces. As a result, this is a huge step forward for the evolution of portable sensors that may be worn on the body.

Due to their high electrical conductivity and wide specific surface area, graphene and graphene derivatives are ideal for the precise and selective detection of target biomolecules via processes such as protein adsorption. In addition, graphene’s strong catalytic activity towards tiny biomolecules such as H_2_O_2_, NADH, and others makes it a promising material for enzyme-based biosensors such as those used to detect glucose and ethanol.

### 6.4. Wearable Biosensors

**Figure 15:**
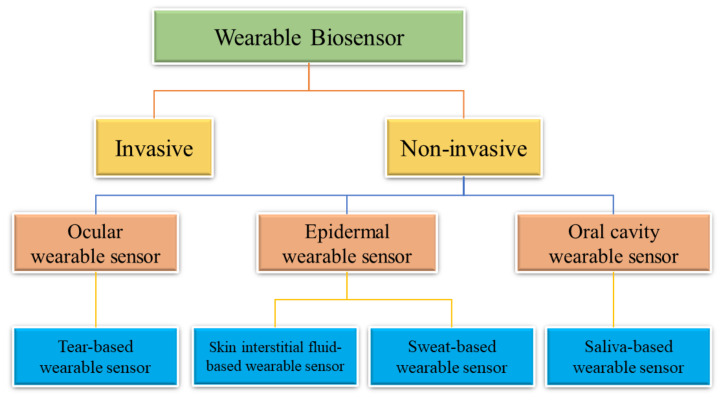
Wearable biosensor classification.

Non-invasive and invasive sensors may be used to measure physiological data, as shown in Figure 15. It is necessary to access bodily fluids by injection or incision for invasive sensors (also known as intrusive sensors). Certain conditions, such as diabetes, cardiovascular disease, and medication effectiveness monitoring, benefit from continuous monitoring of parameters utilising wearable monitors such as glucose monitoring, athletes’ fitness monitoring, oxygen saturation, and cholesterol tracking. Invasive sensors, such as those that require the use of bodily fluids such as blood or serum, are not appropriate in these situations. Because needles have been mishandled, blood contamination is also quite likely [388]. As a result, non-invasive sensors are more appealing and less uncomfortable for the user since they do not need the user to be injected or cut open. This includes saliva, perspiration, tears and skin interstitial fluids [388,389].

**Figure 16:**
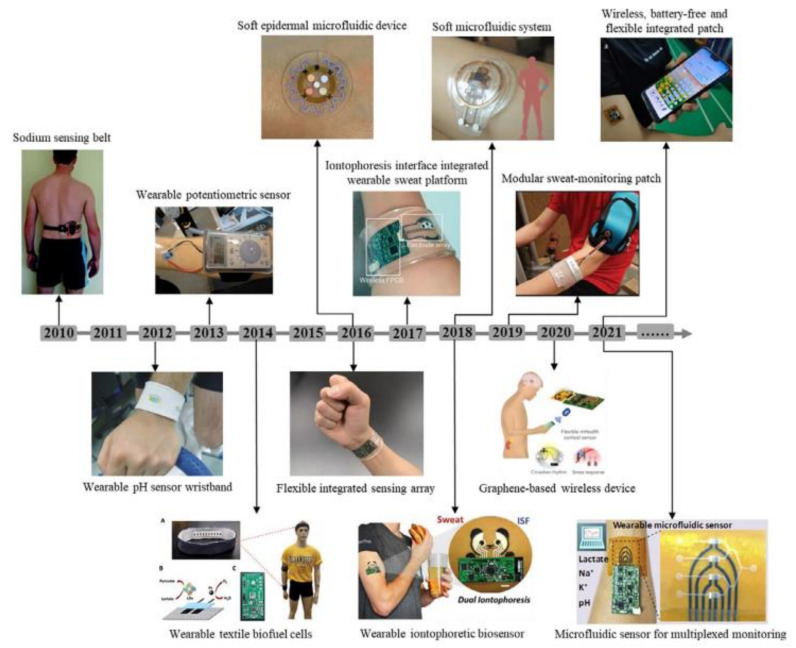
Over the last decade, examples of integrated wearable sweat sensors have been created. (Reproduced from [404] with permission. Copyright 2021, Elsevier, Amsterdam, The Netherlands).

Signal transmission, signal processing, sensing and sweat collection should all be integrated into a full wearable sweat sensor. Most wearable sweat sensors, in reality, need laboratory equipment for data processing, preventing them from being used in real time or in the field. Advanced electronic technologies, such as wireless communication and wearable microcircuits, have been used to overcome these restrictions. More completely integrated sweat sensors have been produced with the combination of a signal processing circuit system and a flexible sweat-sensing interface (Figure 16). Initially, integrated wearable sweat sensors were created by integrating a paper/textile -based sweat management system, a flexible sensing interface, and a tiny device [390,391]. Schazmann et al., for example, developed a sodium sensor belt that included a sweat pump cloth, sodium ion selective electrode, and potentiometer [391]. Wang et al. [392] used tattoo-based solid-contact ion-selective electrodes and a multimeter to create a wearable sweat potentiometric sensor. Wearable pH sensors may be created by attaching an ionogel-coupled microfluidic system to a wrist band using a simple colorimetric technique, allowing for visual sweat pH monitoring without the need of an electronic device [393]. Colorimetric sweat sensors, however, are often insufficient for achieving quantitative and high sensitivity analysis. Because of this, further studies have focused on building an integrated wearable sweat sensor using a small circuit board with wireless signal transduction and fast signal processing. With the help of an electrochemical analyser-on-a-chip, USB battery charger, voltage regulator, microcontroller, and Bluetooth wireless module all powered by a rechargeable button cell battery, Wang et al. developed a tiny printed circuit board for use with tattoo-based potentiometric sensors [394]. Gao et al. took use of a commercially available integrated circuit technology [395] that combines amplification, signal processing, and wireless transmission on a flexible printed circuit board. A completely integrated sweat sensor array was built by combining the flexible printed circuit board and flexible sensing array, and the final findings were shown on a phone using a special mobile application. An acceptable arrangement of microfluidic sweat handling, sensitive element, tiny circuitry, and communication components was used to create several prototypes of integrated wearable sweat sensors [396,397,398,399]. With these integrated wearables sweat sensors, iontophoretic electrodes may be used to trigger sweat secretion. Rogers et al. included a Near Field Communication (NFC) module with epidermal electronics, making it possible to communicate wirelessly with a smartphone that supports NFC [400]. Wearable sweat sensors that include NFC chips into a microfluidic-based sweat system were then developed [401]. Wearable sweat sensors that combine a microchannel with an electrode, a flexible circuit, and an adhesive layer have been suggested. The flexible circuit board with an NFC chip may wirelessly transfer data and is powered by a smart phone [402]. Sweat sensors without printed circuit boards or batteries are smaller and lighter than those that need them. It is also possible to use microscopic sweat sensors in water circumstances after encasing them in a waterproof covering [401,402]. Liu et al. have created a compact integrated sweat patch that links a flexible screen-printed electrode to a flexible circuit board with a conductive bonding pad. The flexible circuit board’s NFC module enables wireless power harvesting and data exchange with an NFC-enabled smart phone [403]. Unfortunately, the distance between the patch and the smart phone limits NFC-based sweat sensors. The aforementioned integrated wearable sweat sensors now provide outstanding prototypes with wanted capabilities; nevertheless, several obstacles must be fixed before they become a reality. Wearable sweat sensors are expected to progress further in terms of integration, reliability, wearable comfort and response speed, as a result of multidisciplinary research that includes novel materials, sophisticated electronics, and high-end manufacturing.

**Figure 17:**
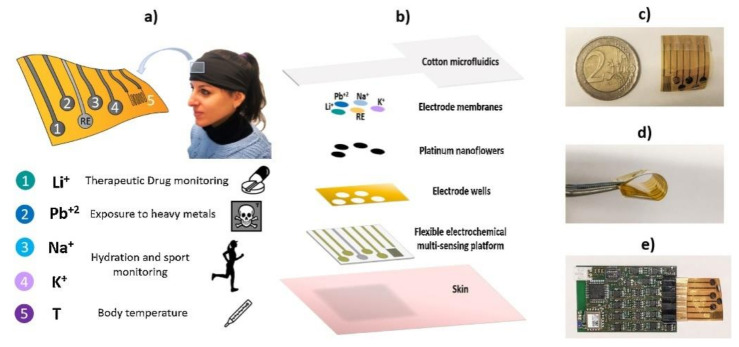
Overview of the sweat-monitoring wearable multi-electrode system: (**a**) medical application; (**b**) the custom made and adaptable electrochemical multi-sensing system; (**c**) the flexible electrochemical multi-electrode platform; (**d**) bending test; (**e**) example of interface with read-out electronics. (Reproduced from [405] under common creative License. Copyright 1969, Elsevier, Amsterdam, The Netherlands).

Despite the enormous growth in sweat sensing and wearable technology over the last several years, a number of obstacles remain, including inadequate sample collection, separate sampling and processing, limited multi-sensing capabilities, and materials toxicity. The wearable multi-electrode device proposed by Francesca Criscuolo et al. [405] addresses some of these concerns. The repeatability and biocompatibility of the sensing technology are achieved by the use of one-step electrodeposited platinum nanostructures. An ionic–liquid junction serves as a stable reference electrode (RE), and four electrodes may be used to simultaneously sense different analytes. A temperature sensor is also included in the platform’s flexible design. New sweat samples are brought to the sensing area constantly, while the already-tested samples are disposed of. It has been shown that this technology’s excellent analytical performance can be used in a variety of different applications, including the monitoring of therapeutic drugs in the treatment of mental illness, the detection of heavy metal contamination with Pb^2+^, and the tracking of athletes with K^+^ and Na^+^. The sensors provide linear responses in synthetic sweat across the clinically relevant ranges. A mannequin is utilised to assess reversibility and selectivity in a simulated wearable scenario. Finally, we were able to monitor the levels of potassium and salt in the blood of five healthy volunteers as they exercised. As shown by Pearson coefficients of 0.97 and 0.81, the in situ readings are reliable (Na^+^ and K^+^, respectively). To develop non-invasive m-Health monitoring devices, this wearable platform represents an important step forward because of its high biocompatibility, selectivity, and accuracy in sample handling. This will help researchers better understand physiological parameters and the clinical requirements for each individual. As seen in Figure 17, a healthcare application for the wearable multi-sensor system is shown. A total of four ion-selective electrode (ISEs) with noble metal nanostructure SCs comprise the platform. It is possible to electrodeposit these materials in only one step (less than 4 min) and achieve outstanding potential responsiveness and stability (capacitance values of 195.3 ± 96.8 μF), as well as non-toxicity and high biocompatibility. It is therefore a viable and safe alternative to the more often utilised conductive polymers and carbon-based nanostructures.

**Figure 18:**
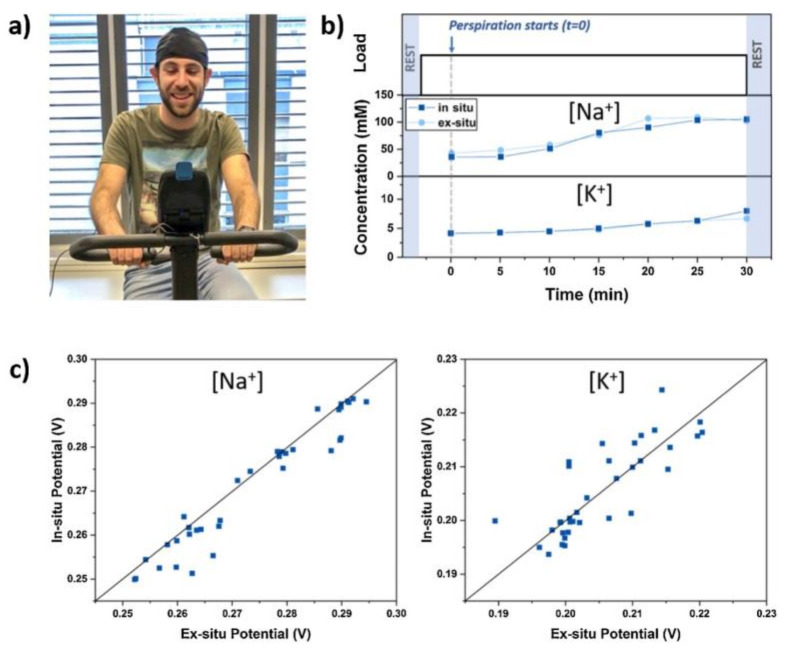
(**a**) Human participants were tested on the wearable platform using this experimental arrangement. (**b**) Thirty minutes after sweating began, the potassium and sodium levels in a volunteer’s sweat were measured using electrolyte analysis. (**c**) Analysis of the sweat of a volunteer after 30 min of indoor cycling to compare in situ and ex situ measures of potassium and sodium. (Reproduced from [405] under common creative License. Copyright 1969, Elsevier, Amsterdam, The Netherlands).

Figure 18a depicts the set-up for the experiment. Comparisons are made between in situ and ex situ measures. For the ex situ measurements, cotton pads are attached and removed every 5 min, and the sample interval is set to 5 min. To ensure that the in situ and ex situ data could be compared, this method was used. However, the system may also be utilised for continuous measurements, as shown by the mannequin’s reversibility and selectivity tests, which show the system’s tremendous potential stability. In addition, the hydrophobic nanostructures of the SCs, which hinder the development of a water layer, were extensively studied. Figure 18b depicts the Na^+^ and K^+^ concentrations in a blood sample taken from one of the subjects. As predicted, the concentrations of sodium and potassium are rising with time. As dehydration progresses, water is lost, resulting in a rise in sweat ionic concentrations. The other participants showed similar patterns. With regard to personal physiological variability and physical training, the little discrepancies may be explained. Ex situ values and in situ values are highly correlated in all subjects. Scattered plots, as seen in Figure 18c, support this finding even further. It is clear that the potentials measured in situ and ex situ have a strong relationship. Na^+^ and K^+^, for example, have Pearson values of 0.97 and 0.81, respectively. They demonstrate the high precision of the proposed flexible SC technology and the effectiveness of fluidics in sweat management, opening the path for biocompatible and repeatable wearable multi-sensing systems to be produced.

#### Wearable Biochemical Sensors for Sweat Analysis

Biochemical, biophysical, and kinematic data generated by the body’s normal physiological processes may be measured non-invasively using new types of skin-interfaced wearable sensors. By utilising the integration of multimodal sensors, traditional wearable biosensors, developed in wrist-worn, chest-strapped, and apparel-integrated forms, allow for quantitative evaluation of physiological parameters in continuous modes of operation. Current wearable technologies [406] allow for remote monitoring of health, performance, environmental safety and daily activities; however, they are unable to characterise important metabolic processes, which are necessary for developing a comprehensive understanding of health, nutrition and wellness status. Intravenous blood testing and the use of large, expensive lab instruments are both standard ways for determining body chemistry. In addition to blood, other non-invasive biofluids including tears, saliva and perspiration may be used to analyse biomarkers and provide remote health monitoring outside of a laboratory environment [407]. Micronutrients, nucleic acids, proteins, hormones, electrolytes, metabolites, and exogenous substances are all present in sweat [408,409]. Sweat analysis approaches in the lab have included a mix of strap-based flexible tubes, absorbent pads, wicking polymers, and sticky tapes for collection, with separate laboratory apparatus for examination. For medical diagnoses or regulated sports performance monitoring, this strategy is adequate; nevertheless, the necessity for skilled workers, the use of expensive equipment, and sophisticated sample processes limit its applicability to highly controlled laboratory conditions. Biochemical sensors, flexible/stretchy electronics, and material science advances have laid the groundwork for a new class of skin-interfacing wearable platforms that can analyse sweat’s biomolecular composition and dynamics in either a continuous or intermittent manner without the need for external instrumentation [410,411,412]. In terms of improved measurement precision, deployment methods, and integration with other body-worn sensors, the ramifications are enormous. With real-time assessment of sweat components, either alone or in combination, as well as its underlying dynamic metabolic activity, it is possible to obtain a better understanding of how the human body works. Wearable sweat biosensors have made significant progress over the last several years, with special attention on biosensor ideas that will guide the development of future platforms. Wearable sweat sensors have been examined in the context of skin-interfaced devices [413,414,415,416] or in terms of particular applications [417,418], material systems [419,420], and manufacturing processes [421]. Furthermore, this viewpoint analyses the quick and continual advancement of wearable sweat sensors via the most sophisticated implementations that overcome the basic problems preventing broad application. The essential sensing techniques and manufacturing constructions that underlie these wearable sweat systems are summarised in a brief introduction section. In the second part, instances of current methodologies are used to classify power management, analytical performance and fluid handling difficulties. There is a discussion of efforts to boost overall usefulness and commercialization possibilities, and biochemical sensor developments will be critical.

## 7. Wearable Sweat Sensor Technology

Wearable sweat-based biochemical analysis systems must be able to handle a wide variety of scenarios, from passive sweat collecting to strenuous activity. The humidity or warmth of the environment (tropical vs. desert climate) may also differ, as well as the presence of damp environments (e.g., aquatic sporting activities). In order to allow for continuous sweat sampling and collecting, these demanding-use cases need meticulous designs that create and maintain a solid conformal contact with the epidermis. Chemical sensors with essential performance criteria, such as operational stability, accuracy, power efficiency, selectivity, sensitivity, and data transmission mechanism are likewise subject to such demands. Colorimetric, electrochemical, and hybrid sweat sensors are three new types of wearable sweat sensing platforms that emphasise critical methodologies for addressing these operating aspects.

### 7.1. Colorimetric Sweat Sensors

Low-modulus, breathable elastomeric platforms with embedded microfluidic passages are used for the collection and storage of sweat in epidermal microfluidic (“epifluidic”) devices. The quantitative measurement of sweat elements of interest is made possible by combining colorimetric [422] or fluorescence [423,424] assays. Such devices use natural sweat gland pressure to define sweat rate while also routing sweat to discrete chambers where sweat components mix with specialised chemical reagents to provide a unique optical signal matching to a desired analyte concentration. With recent findings revealing measurement of targets such as sweat lactate, creatinine, urea, glucose, pH and chloride [425,426,427,428], smart phone-based picture capture and colour-based analyses provide a straightforward, affordable analytical method.

### 7.2. Electrochemical Sweat Sensors

**Figure 19:**
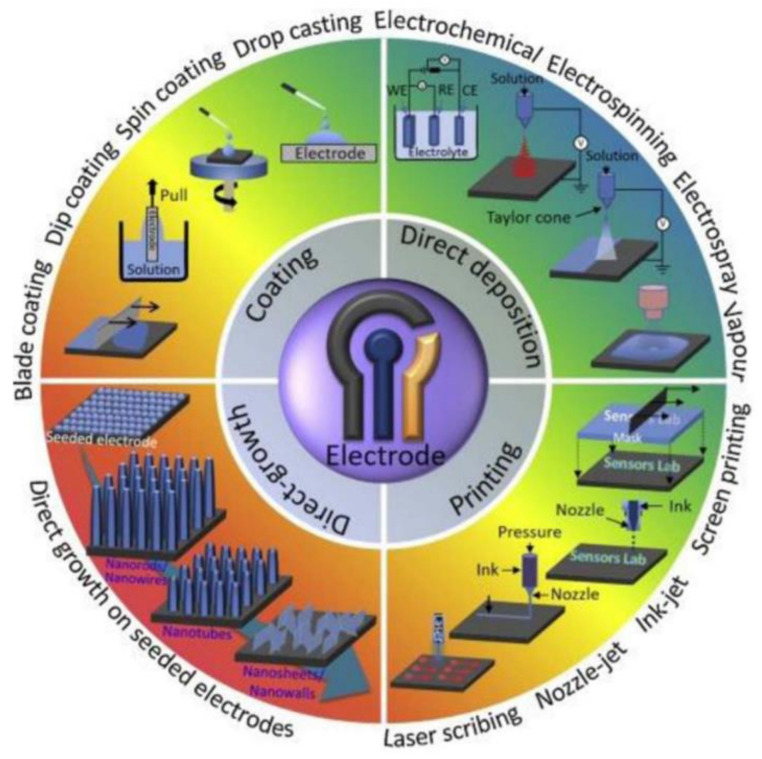
Graphene-modified electrode manufacturing techniques are shown schematically in this diagram. (Reproduced from [440] with permission. Copyright 2018, Elsevier, Amsterdam, The Netherlands).

Sweat biomarker concentrations have been demonstrated to dynamically alter in response to health, stress, and food [429], making real-time monitoring of sweat biomarkers essential. Conductometric, amperometric, potentiometric, and voltimmetric measuring methods are used by skin-interfaced electrochemical sensors to allow for continuous monitoring of target analytes in sweat [430]. For real-time sweat analysation, such techniques give electromagnetic currents corresponding to analyte quantities with high precision as well as quick response times and low power consumption, allowing for miniaturised sensor designs that can be integrated into wearable systems that use onboard memory modules and wireless connectivity to transmit information. Numerous studies [431,432,433,434,435,436] have evaluated graphene-based sensors and biosensors from various perspectives in the past two years. Sun et al. [437], for example, assessed the most recent graphene-based sensors for human health monitoring. The analytical performances of sensors and biosensors based on graphene-related materials in the area of clinical, environmental, and food sciences are discussed in another study [438]. A review of contemporary electrochemical sensors and biosensors for biomarker detection based on graphene and graphene-like materials was also published [439]. The electrodes used in graphene and graphene-based materials sensors and biosensors play a significant role. There are already a variety of ways for producing and modifying electrodes (Figure 19) [440]. In their study, Cinti and Arduini [441] discussed several methodologies for the manufacture and implementation of graphene-based screen-printed (bio) sensors. They also offered a number of ways for modifying screen-printed electrodes, the majority of which could be used to change other electrodes as well.

### 7.3. Hybrid Sweat Sensors

There have been recent developments in smart technologies [442] that emphasise a hybrid strategy that incorporates electrochemical and optoelectronic smart sensors into a single analytical framework for the wireless and rechargeable batteries multimodal analysis of biological markers in consistent and spot check operational mode (e.g., sweat rate, glucose, ascorbic acid and cortisol). A combination of colorimetric lateral flow immunoassays, luminescence analysers for glucose and ascorbic acid, and impedance-based sensors for sweat rate and galvanic skin response may be employed to measure the activity of the sweat glands in this dual-sensing system. Field testing shows that these skills may be used to measure physiological markers related to physical and mental stress over many days. The deployment options that come from this hybrid method hold a lot of potential for long-term continuous and intermittent monitoring of physiological indicators and situations.

## 8. Detection of Analytes from Sweat Using Graphene

### 8.1. Glucose

There are a number of different enzyme-based glucose sensors that may be used to detect levels of glucose in the bloodstream, such as the enzyme-based glucose biosensor (EGBS). Clark and Lyons developed the first enzyme-based glucose biosensor in the 1960s, which utilised the enzyme glucose oxidase (GOx). Biosensors often employ the enzyme GOx. It prefers glucose over other substrates. Due to its affordability and ability to withstand larger temperature, pH, and ionic strength fluctuations, it is the favoured material [443,444,445]. Glucose oxidation or reduction may be measured using direct electrochemistry, which is a fast and inexpensive method. The oxidation of glucose on an electrode of metal has three primary drawbacks: The comparatively slow kinetics of glucose electro-oxidation on standard electrodes limits the sensitivity of glucose sensing. Non-enzymatic sensors for glucose have a poor selectivity due to the oxidation intermediates of glucose and the adsorbed ions of chloride that limit the activity of noble metal electrodes. Other polysaccharides and biological interfering chemicals may also be oxidised in the potential range of glucose oxidation. A variety of nanomaterials with distinct features are being developed currently in an effort to give new prospects for the fabrication of innovative non-enzymatic glucose sensors.

#### Glucose-Sensing Mechanisms

The fundamental process of glucose mutarotation is critical to understanding the glucose oxidation mechanism. The electrooxidation of glucose on the electrode surface modified with nanomaterials that function as catalysts has been explained by two different methods. A catalyst that participates in electrochemical processes is known as an electrocatalyst. Catalyst materials change and speed up chemical processes without being consumed themselves. Electrocatalysts are a kind of catalyst that works at electrode surfaces or may even be the electrode surface. For example, an overall half-reaction may be facilitated by the use of electrocatalysts, which can speed up the transport of electrons between electrodes and reactants. For example, platinum nanoparticles or a platinum surface may be used as an electrocatalyst. It can also be homogenous, such as a coordination complex or enzyme.

- The mutarotation of glucose

**Figure 20:**
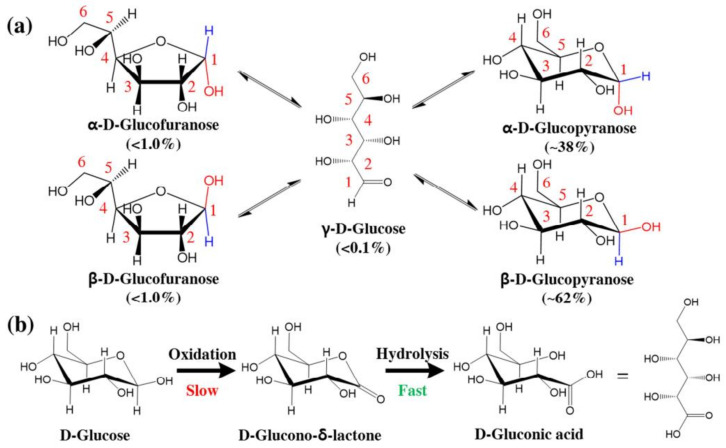
(**a**) Molecular patterns of different D-glucose isomers. (**b**) The whole process of oxidising glucose. Slow hydrogen abstraction and oxidation transform D-glucose into D-glucono-δ-lactone, which is then rapidly hydrolysed into D-gluconic acid. (Reproduced from [452] with permission. Copyright 2018, Elsevier, Amsterdam, The Netherlands).

The existence of glucose is in different structural forms: α- glucose (α-G), βglucose (β-G), γ-glucose (γ-G). There are three distinct isomeric forms of glucose. It is also known as α-glucose (α-G), β-glucose (β-G), γ-glucose (γ-G). α-G, β-G and γ-G are in a state of equilibrium at RT (room temperature) in water at a ratio of 37:63:0, respectively [446]. Figure 20 depicts glucose in its most stable cyclic form i.e., an open-chain aldehyde structure γ-glucose in aqueous solutions cycles via hemiacetyl bonds to ring structures pyranose and furanose in water. These anomers may be categorised into α and β and depending on the hydroxyl group linked to C-1. As shown in Figure 20, the pyranoses (α, β) interconvert to create an equilibrium by the hydrolysis of an intermediate made of γ-glucose, which is an acid–base catalysed. This is referred to as mutarotation. It often takes up to 2 h for this sluggish process to reach equilibrium at room temperature [447,448]. pH and temperatures have a major role in this process [446,447,449]. Step 1 of the non-enzymatic glucose sensor is to remove a hydrogen atom from C-1. β-glucopyranose is preferable over α-glucopyranose in this case. GOx and glucose dehydrogenase (GDH) enzymes, which oxidise glucose, have similar behaviours. Dehydrogenated glucose is immediately oxidised to gluco-δ-lactone, which is then promptly hydrolysed to generate gluconic acid with a fixed rate of 10^−3^ s ^−1^ at pH 7.5, regardless of the anomeric forms present [450,451].

- Chemisorption model

Analyte binding to electrode’s active areas, where a sufficient bond is made with the adsorbate, is the most common method of electrocatalysis, as indicated by the chemisorption model [453]. Bonds are broken, and intermediates are produced after the reactant adsorption process. The contact between the electrode and the products weakens, resulting in product desorption from the electrode’s surface. This is referred to as the chemisorption model [453,454]. The closer the glucose molecule is to the electrode, the more C-1 and its hydrogen atom interact with it. Adsorption occurs on the electrode surface as a result of C-1 dehydrogenation. This results in glucose electrooxidation.

- IHOAM model

**Figure 21:**
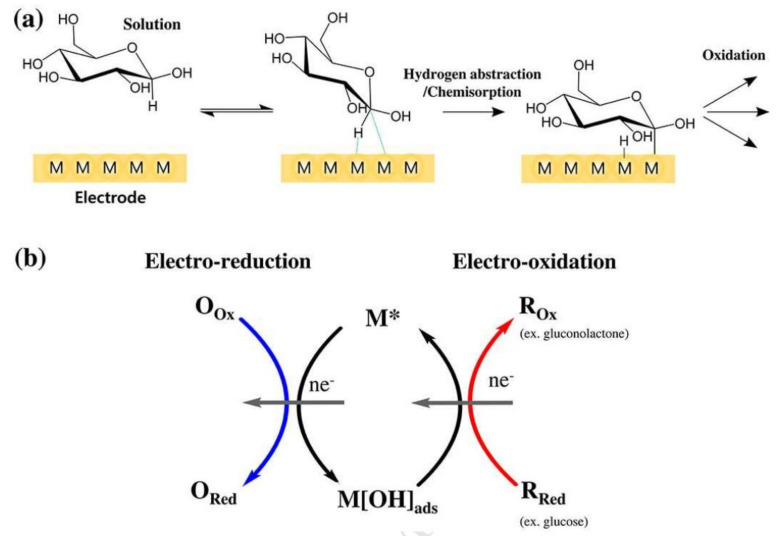
(**a**) The mechanism of oxidation of glucose. (**b**) The effect of reactive species in the fast conversion of glucose into glucono-δ-lactone. (Reproduced from [458] under common creative License 4.0).

The “Incipient Hydrous Oxide Adatom Model” (IHOAM) suggested by Burke is a different model used to describe the oxidation of glucose. In this instance, the hydroxide layer is pre-oxidation produced. The metal centres have coordination numbers that are very low, which are used to create this OHads pre-monolayer [455]. Using a gold or platinum electrode as an example, the IHOAM model still remains true. In terms of redox processes involving lower and higher oxidation numbers, glucose oxidation on transition metals may be described [456]. Various varieties of non-enzymatic electrocatalysts are available. In addition to platinum, gold and other precious metal oxides, alloys and carbon-based compounds are included in the list of metals. A bond is formed between the analyte and the electrodes when d-electron and d-orbitals of the metal substrate adsorb onto the electrode surface, resulting in the creation of a bond with the analyte. In the adsorption process, the electrode shape is very important. It is thought that the processes of hydrogen abstraction and organic species adsorption happen at the same time. That is, hemiacetalic hydrogen elimination and organic analyte chemisorption happen at the same time [457]. The use of sites transition metal to explain the adsorption process on electrode surfaces is a success. However, it fails to explain the process involved in hydrogen radicals’ oxidative action. The improved catalysis of platinum group metals (iridium, ruthenium, platinum, and palladium) with gold seems to be best explained by the IHOAM model. This model may also be used to explain glucose electrocatalysis on Cu and Ni electrodes; thus far, in place of a pre-monolayer synthesis, the oxidation state shift of metal hydroxide is more important in this case. The oxidation of glucose in its pure form is an extremely sluggish process that produces no useful current. Hence, for glucose oxidation, electrocatalytic reactions are essential. As a result, scan rate-dependent voltammetry does not conform to direct glucose oxidation, suggesting a non-diffusion-controlled mechanism. Figure 21a shows the process through which glucose is oxidised via chemisorption. Prior to being further oxidised, the reactant (glucose) is chemically adsorbed by a coordinated process of hydrogen abstraction and chemisorption of reactive intermediate onto the metal electrode surface. Figure 21b provides the novel mediator model involving adatom and hydrous oxide. The rapid electro-oxidation of glucose into glucono-δ-lactone is thought to be facilitated by the electrode’s reactive hydrous oxide (OHads) layer.

- Graphene-based electrochemical sensing of glucose

After the discovery of graphene through mechanical exfoliation in 2004 and the Nobel Prize in Physics in 2010 for its remarkable properties, the saga of graphene has come to a conclusion [459]. Graphene is the only material known to exist that is made up of only one layer of carbon atoms connected together by hybrid bonds that form hexagonal honeycombs. A unique feature of graphene is its 2p-orbitals, which make up the state bonds [460]. The unique qualities of graphene, including as its ability to modify electrical parameters such as charge concentration, mobility, and density, as well as its band gap and electron transfer rate, have made it a viable candidate for use in electrochemical sensors. Graphene is a great material for electrodes because of its outstanding features, which include a large surface area, a large potential window, high flexibility and durability, and low charge transfer resistance. When compared to carbon nanotubes, graphene has considerable benefits such as a large surface area, cheap cost, simplicity of production, and safety [461]. Because of a lack of some transition metals such as Ni, Fe, and other impurities, graphene has a high purity [462]. Graphene is an appealing material for enzyme-based biosensors such as glucose and ethanol biosensors due to its high transference of electrons for certain enzymes and particularly very high catalytic performance towards tiny substances such as H_2_O_2_ and β-nicotinamide adenine dinucleotide (NADH). As a biosensor is developed, enzyme electrochemistry, or electron transfer between enzyme and electrode directly without intermediates, is essential. As a result, direct electrochemistry with ordinary electrodes is difficult since many redox enzymes have their active centres in hydrophobic cavities of molecules [463,464]. Because of its large specific area and excellent electron transport, functionalized graphene facilitates direct electrochemical processes between electrodes and enzymes [465]. Because of their large surface area, the electrodes of graphene possess a large loading of enzyme, which improves the performance of biosensors that are derived from graphene [465]. Graphene is a potential material for oxidase biosensors because of its strong electrocatalytic activity towards H_2_O_2_ and exceptionally good direct electrochemistry of GOx. Nanomaterials are a feasible choice for the fabrication of high-performance non-enzymatic glucose sensors due to their ultrahigh conductivity, robust electrocatalytic activity, large surface-to-volume ratio and excellent transference of electrons between the electrocatalyst and conductive electrode substrate [466]. Polyethyleneimine-modified electrodes were employed in the first graphene-based glucose biosensor, which showed an excellent repeatability, great stability and wide linear glucose response [463]. The biosensors of glucose that employed biocompatible chitosan to spread graphene had good sensitivity and long-term stability, according to the researchers [465]. It has been reported that a glucose sensor system relying on CR-GOx (chemically reduced graphene oxide) has enhanced amperometric responses for monitoring glucose, with a wide linear range (0.01–10 mM) and a 2.0 µM limit of detection [467,468]. Glucose sensors (enzymatic) based on nitrogen-doped graphene showed improved electrochemical performances [469]. The electroanalytical activity of a graphene/AuNPs/chitosan composite film-based biosensor for H_2_O_2_ and O_2_ has been described [470]. This is owing to graphene and metal nanoparticles’ excellent electrical conductivity, wide surface area, and synergistic impact.

**Figure 22:**
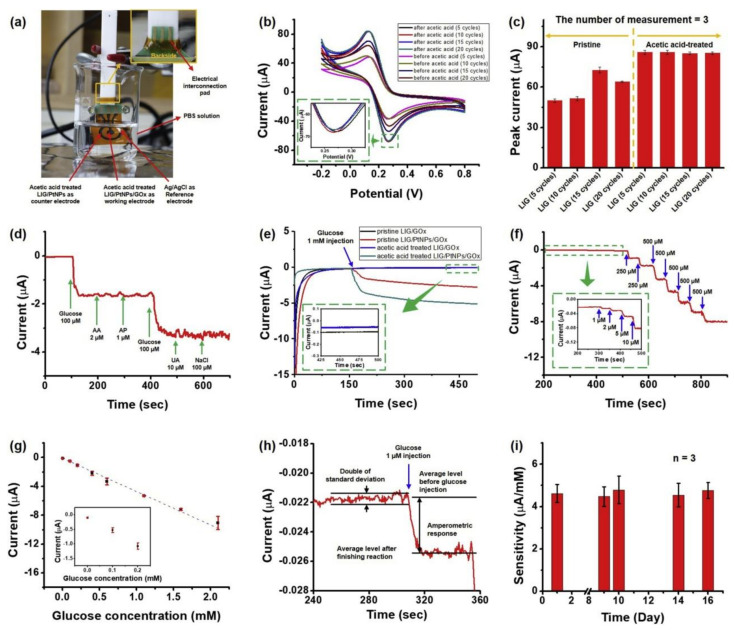
Results of electrochemical characterizations. (**a**) different electrochemical in vitro characterisation test configurations, (**b**) CV conducted in K_3_[Fe(CN)_6_] solution to compare the redox properties of pristine LIG with PtNPs and acetic acid-treated LIG with PtNPs at a scan rate of 50 mV/s, (**c**) cyclic voltammogram’s cathodic peak current, (**d**) urrent response of glucose and interferences such as AP, AA, NaCl, and UA, (**e**) current responses of different LIG−based electrode samples, (**f**) The current response of the various glucose concentration from ultra-low glucose levels. (**g**) Linear regression functional curve. (**h**) Current response under 1 μM glucose injection. (**i**) Daily variation of the sensitivity of the as-produced acetic acid-treated LIG/PtNPs/GOx electrode. (Reproduced from [471] with permission. Copyright 2020, Elsevier, Amsterdam, The Netherlands).

Hyosang Yoon et al. [471] recommended altering the surface of the laser-induced graphene electrode with acetic acid using a simple dipping method. The carbon–carbon bond ratio was improved, resulting in increased conductivity and decreased sheet resistance. In addition, the unique properties of LIG prevented nanoparticle agglomeration during electrodeposition, allowing for the stable and uniform dispersion of highly catalytic Pt nanoparticles (PtNPs) on LIG. The sweat glucose biosensor was then put onto a LIG/PtNPs electrode. The sensitivity of the LIG/PtNPs electrode is 4.622 A/mM, with a signal-to-noise ratio of 3 and a dynamic linear range of 2.1 mM. The results of many electrochemical characterizations are shown in Figure 22 [471]. Figure 22a shows different electrochemical in vitro characterisation test configurations. Figure 22b shows a cyclic voltammogram that was conducted in K_3_[Fe(CN)_6_] solution to compare the redox properties of pristine LIG with PtNPs and acetic acid-treated LIG with PtNPs according to electrodeposition cycles, with a scan rate of 50 mV/s. Figure 22c shows the cyclic voltammogram’s cathodic peak current. According to the optimum applied potential, the current response of glucose and interferences such as AP, AA, NaCl, and UA is shown in Figure 22d. Figure 22e shows the obtained current responses of different LIG−based electrode samples compared. Figure 22f shows the current response of the various glucose concentration from ultra-low glucose levels. Figure 22g shows linear regression functional curve. Figure 22h shows current response under 1 μM glucose injection. Figure 22i shows daily variation of the sensitivity of the as-produced acetic acid-treated LIG/PtNPs/GOx electrode.

**Figure 23:**
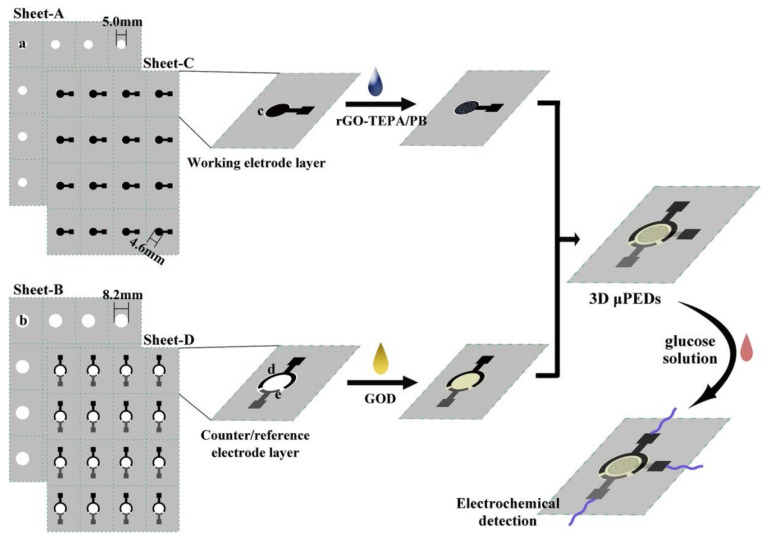
Preparation of a three-dimensional microfluidic electrochemical biosensor based on paper. (**a**) preparation of a three-dimensional microfluidic electrochemical biosensor based on paper, (**b**) 8.2 mm hydrophilic zone, (**c**,**d**) carbon as counter and working electrode, and (**e**) reference electrode Ag/AgCl). (Reproduced from [472] with permission. Copyright 2019, Elsevier, Amsterdam, The Netherlands).

Photolithography and screen-printing methods were employed by Liangli Cao et al. [472] to build a novel screen-printed electrode (SPE) with two layers. A hydrophilic aldhyde-functionalized zone of the electrode, i.e., references and counter, layer was produced for the immobilisation of glucose oxidase. The enzyme-catalysed reaction product, H_2_O_2_ (hydrogen peroxide), may be quantified using an electrochemical sensitive membrane based on rGO-TEPA/PB (rGO-tetraethylenepentaamine)-modified paper electrode layer covered with Prussian blue. As a consequence, this microfluidic electrochemical biosensor based on 3D paper may be used to sense glucose quantitatively. For a detection limit of 25 mM, the proposed biosensor may be utilised to measure quantitative glucose across an ideal 0.1–25 mM linear range. Finally, a 3D paper-based microfluidic electrochemical biosensor was used to detect glucose levels in sweat and blood, and the findings were extremely comparable to those acquired with Roche’s blood glucose metre. Furthermore, the anticipated paper-based 3D electrochemical device showed outstanding anti-interference properties, stability and repeatability, suggesting that it has a lot of potential for monitoring glucose in complex biological fluids. Preparation of a three-dimensional microfluidic electrochemical biosensor based on paper is shown in Figure 23 ((a), 5 mm hydrophilic zone; (b), 8.2 mm hydrophilic zone; (c,d) carbon as counter and working electrode; and (e), reference electrode Ag/AgCl) [472].

**Figure 24:**
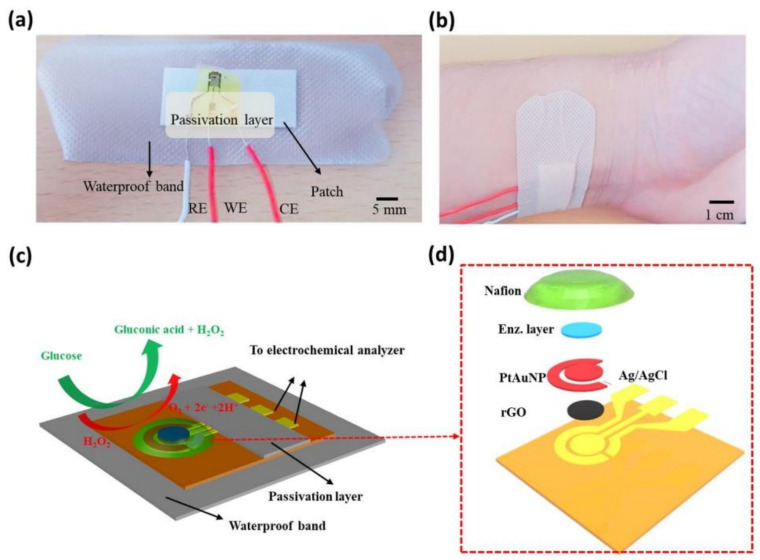
Schematics of the developed wearable glucose biosensor based on sweat. Photos taken with an optical camera (**a**,**b**) of the developed biosensor. Diagram of the whole biosensor (**c**), with an expanded view (**d**). (Reproduced from [473] with permission. Copyright 2018, Elsevier, Amsterdam, The Netherlands).

When it comes to non-invasive sweat glucose monitoring, the patch-based electrochemical biosensor developed by Xuan et al. [473] is the best option yet. High selectivity is provided by an enzyme-based electrode; a miniaturized, low-temperature rGO production technique employing microelectromechanical systems (MEMS) provides reliable analysis of sweat even with very little amounts of sweat, and a low-temperature manufacture approach is simple, low-cost, and reliable. Additionally, it is able to mass produce graphene/metal nanoparticle-based working electrodes in large quantities. Using a sweat glucose sensor as a patch-type sensor for human blood glucose monitoring has great potential. Figure 24 depicts a patch-type MEMS electrochemical biosensor with a flexible architecture that permits great performance even when physically deformed. Figure 24c,d show a schematic representation of the sensing mechanism. A GOx/Nafion layer covers the Au/rGO/AuPtNP electrode. To convert glucose into H_2_O_2_, GOx needs a cofactor that facilitates the reaction. The redox potential measures the produced H_2_O_2_ amperometrically on the working electrode’s surface, allowing the current to be linked to the glucose concentration. O_2_, electrons, and an H^+^ ion will be formed from the H_2_O_2_. To prevent the sensor from delaminating from the skin, a waterproof band is placed beneath the flexible sensor to help in sweat collection.

This century’s pandemic of diabetes is not only impacting adults but also children, regardless of gender or age, and it has become one of the main reasons of mortality among the other noncommunicable diseases (NCDs) [474]. For example, the number of children (0–19 years old) with type-1 diabetes has surpassed one million for the first time (1,106,500), with the United States and India ranking first and second, respectively, with 169,900 and 128,500 afflicted children. As per the IDF (International Diabetes Federation), the number of persons diagnosed with diabetes worldwide is predicted to climb everyday from 425 to 629 million people by 2045 [475]. For health status monitoring, non-invasive or blood-free diagnostics are becoming increasingly popular since they may lower discomfort and various risk factors compared to conventional invasive testing. The non-invasive diagnostic equipment may be made smaller and more wearable so that it can be used for continuous, real-time monitoring. Several researchers have successfully produced a range of designs, including as contact lenses [476], eyeglasses [477], tattoos [478], and wristbands [479], which directly touch the skin and body of a person for non-invasive analysis as wearable sensors. As a result, substrates for wearable sensors must be stretchy, flexible, light, biocompatible, and pleasant to the human skin. Textile [480], paper [481], and synthetic polymer [482] have all been employed as substrates for non-invasive sensor construction. Wearable biosensors have made significant breakthroughs in monitoring an individual’s physiological status, in particular [483]. There are a number of wearable biosensors on the market, but electrochemical sensors for monitoring vital signals through the detection of physiological components in sweat have attracted the most attention [484]. Many individuals have diabetes, which is a chronic condition caused by inadequate insulin production [485]. Because diabetes causes so many serious consequences, the patient’s blood sugar level must be constantly checked. In most cases, diabetes may be diagnosed by examining the blood glucose level. When blood glucose concentrations exceed 126 mg dL^−1^ while fasting, hyperglycaemia develops [486]. Detecting glucose levels in blood, however, is a pain since it requires bleeding with a needle. Moyer et al. [487] convincingly demonstrated the relationship between glucose concentrations in blood and sweat, demonstrating that 300 mg dL^−1^ glucose in blood equates to 0.3 mM glucose in sweat. Because of this, it is predicted that the glucose level may be detected using a sweat sensor for diagnosis of diabetes. As a result, the quantity of glucose in sweat may be used to determine a person’s health. A sensor that does not adapt to the skin and does not have adequate mechanical stability would deform due to the skin’s mobility, making it impossible to reliably monitor pH and glucose levels in sweat [488]. As key markers of biological processes in the human body, the movement of metabolites and nutrients in biofluids may be utilized to predict outcomes, diagnose disease, and evaluate clinical risk and track treatment progress. Deficiencies in nutrient and metabolite circulation (i.e., concentration) are directly linked to health conditions such as metabolic syndrome and cardiovascular disease. The use of wearable sensors in medical technology is critical to the development of customized medicine, which involves monitoring a patient’s health over time. Sweat samples, which contain a wealth of physiological data, might be used to provide non-invasive monitoring [489]. For example, the level of chloride in human sweat serves as a gold standard for the treatment of cystic fibrosis, and the concentration of glucose in sweat is the primary method used to control diabetes [490]. As a biological fluid, perspiration may be used for non-invasive study of the human body. Proteins (e.g., neuropeptides and tumour necrosis factors) as well as tiny molecules (e.g., uric acid and lactate) may be found in sweat. Sweat also includes metabolites and electrolytes (e.g., cortisol, urea and amino acid) [481]. There are biomarkers in sweat that may tell you about your health. Glucose is regarded to be the most important biomarker in sweat since it might indicate the presence of diabetes [491,492]. As a result, diabetes has been ranked as one of the most frequent chronic diseases in the United States. As a result of excessive glucose levels in diabetes patients’ blood, many of their symptoms include blindness, nerve degeneration and kidney failure as well as wounds (non-healing) and renal illness [493]. The food business, clinical diagnostics, individual blood glucose control, biochemistry, and other fields all benefit from rapid and accurate glucose level detection [494]. As a result, the pressing need for diabetes sensors with high levels of stability, selectivity, and sensitivity draws a lot of attention from researchers every year. Selectivity is the driving force behind the development of an enzymatic glucose sensor. Enzymatic sensors, such as modified glucose oxidase, are often employed to measure glucose levels in the bloodstream [495]. The high-performance sensors for sensing glucose using graphene-based materials are tabulated under Table 5.

### 8.2. Lactate

**Figure 25:**
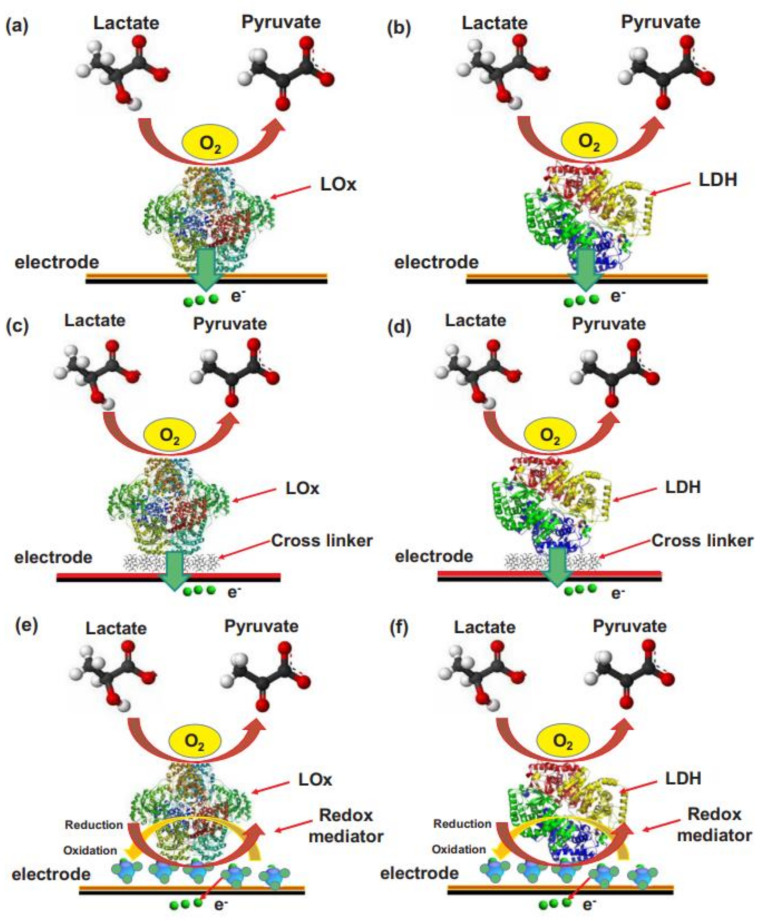
Diagram illustrating direct electron transport in (**a**) LOx, (**b**) LDH, (**c**) cross-linker attached LOx (**d**) cross-linker attached LDH; and for mediated electron transmission in (**e**) LDH and (**f**) LOx. (Reproduced from [555] with permission. Copyright 2018, Elsevier, Amsterdam, The Netherlands).

There are several applications for lactate concentrations outside clinical diagnosis in sports medicine, shock/trauma, and the food business. As an indicator of food quality and freshness, lactate may be utilised in the food business, while in sports medicine, lactate levels are used to assess an athlete’s physical fitness [538]. Lactate determination may also be performed via high performance liquid chromatography, fluorometry, colorimetry, chemiluminescence, and magnetic resonance spectroscopy. These approaches have the disadvantages of being time consuming and expensive, necessitating the use of specialised equipment and skilled labour. Some of these drawbacks may be circumvented by using biosensors. It is easy to use, inexpensive, and provides real-time data capture and quick reaction. Biosensors based on enzyme lactate sensors are the most frequent because of their low detection limit, easy manufacture and mobility as well as their high sensitivity and reliability [538]. Currently, non-invasive lactate sensor applications such as saliva [539], sweat [540], and tears monitoring [541] are popular. The easiest way to measure lactate during exercise is via perspiration [542]. It has been shown that sweat is the best biofluid for measuring lactate levels. This is due to the fact that perspiration can be readily collected and provides physiological data. Peripheral occlusive artery disorders and soft tissue injury may be accurately assessed using sweat lactate as a biomarker [543]. LOx and LDH are two enzymes often utilised for lactate tests. Analyte concentrations may be linked with their electro-reactive reagent consumption, which generates an electro-reactive species that can be monitored. In the following, we discuss the enzymes’ physicochemical and biological characteristics. Nanomaterials have been used in lactate biosensors, and the reaction involving better electron transport has been significantly improved [544]. Aerobic lactate oxidation to PA is catalysed by LOx, generating hydrogen peroxide (H_2_O_2_). An electrochemical reaction restores the oxygen concentration by oxidising it, and a proportionate response is obtained [545]. LOx may be found in a variety of bacteria, including Pedi coccus, Aerococcusviridans, and Mycobacterium smegmatis, as well as viruses and cellular organisms. Their stability and activity may vary with temperature and pH, according to various sources. It uses FMN (flavin mononucleotide) as a cofactor to catalyse the oxidation of hydroxyl acids in a process involving long chain a-hydroxyl acid oxidase, flavocytochrome b2, L-lactate monooxygenase, LOx, glycolate oxidase and L-mandelate dehydrogenase, which is a member of the FMN family. Oxygen is used as an oxidant to form oxygenase, which is an oxidoreductase enzyme. Stability of this enzyme in the pH range of 4–9 depends on the source [546]. In the presence of a particular enzyme, electrochemical biosensors might be a useful tool for detecting lactate, which can be directly translated into electrical signals [547]. The electron transfer mechanism and enzyme immobilisation approach are essential to the functioning of this technology. Lactate electrochemical sensing is a two-step process that includes the following two phases. Biocatalysts are immobilised on a supporting substrate near the electrode surface in electrochemical biosensors. Immobilization techniques, the kind of the biocatalyst utilised, and the other adsorbing species via electron transfer (ET), additives and mediators all have a role on the biocatalyst’s response. Several methods for immobilising enzymes exist, each with its own advantages and disadvantages. A cross-linking agent, dialysis membranes, polymeric films, physical adsorption, entrapment, covalent coupling, and integration into the bulk of a carbon composite matrix are a few examples [548]. ET’s framework is capable of performing responses that are both selective and specific [549]. During ET, electrons are moved from the enzyme’s active site to the electrode. The immobilisation medium should be developed to improve electron transport in order to speed up the bio-molecule-to-electrode process [550]. The construction of electrodes of enzymatic sensor to allow for the process of ET directly or enzymes modified with mediator in the detection of L-lactate has used a variety of methodologies. Cellular processes are fundamentally influenced by protein-mediated ET. ET between the electrode and the substrate is made possible by the redox enzyme acting as an electro-catalyst [551]. Biosensors with operating potentials near to the enzyme’s redox potential are known to be more selective because they are less susceptible to interference reactions. Direct (mediator-free) ET may catalyse a number of enzymes. As demonstrated in Figure 25a,b, this process involves both electrotransfer (ET) from electrode to substrate molecule and catalysis throughout the whole process. Figure 25c,d demonstrate how the cross-linker connects the enzyme’s active site to the transducer surface. To summarise, in the bio-electrocatalysis without mediators, the electrode itself serves as a substrate, and enzyme transformation does not constitute a distinct reaction. Charge is transported between the enzyme’s active redox centre and transducer through diffusional electron mediators [552]. Anti-interference mechanisms are built into the electrode to prevent redox reactions from interfering with other processes, such as photosynthesis. Polymeric or perm selective membranes, compounds used in redox reaction and other compounds of transition metal are some examples of important utilizing materials [553]. Linearity, reduced detection limit, superior electron transport, selectivity, sensitivity and better stability have been shown to boost sensor performance. Interfering electro-active species are repelled by the combination of matrices and mediators with a recognition layer of nanoparticles (NPs), resulting in stable lactate quantification. As illustrated in Figure 25c,d, cross-linkers have been found to exclude interference, resulting in increased stability, foul prevention, and protection of the enzyme structural composition [554]. Figure 25e,f depict the mechanism of ET mediated by lactate’s two main enzymes (LDH and LOx).

**Figure 26:**
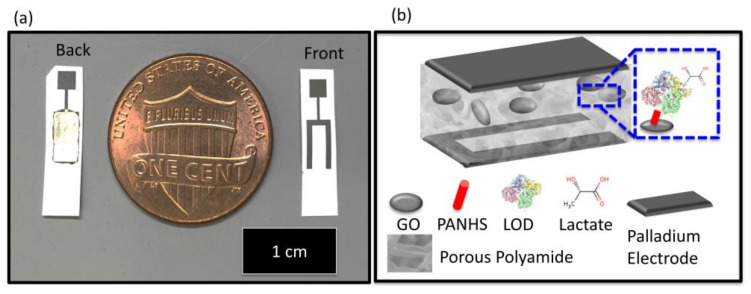
(**a**) Lactate sweat sensor optical visualisation. (**b**) The electrochemical affinity-based lactate detection technology using graphene oxide across a membrane is shown schematically. (Reproduced from [556] with permission. Copyright 2020, Elsevier, Amsterdam, The Netherlands).

Low volume sensing platforms were developed by Kai-Chun Lin et al. [556] using graphene oxide (GO) nanosheets as a transduction element. A porous polyamide (PA) membrane’s holes were filled with GO nanosheets after vacuum was applied across the electrode’s sensing region. Because of the hydrophilic nature of the PA membranes, the whole sensing zone may be covered with less than 5 µL of fluid. Three-dimensional (3D) sensing is possible because of the placement of distributed GO nanosheets between vertically oriented electrodes. Cortisol may be detected across the membrane substrate using 3D electrodes rather than 2D electrodes, as previously described [557]. As can be shown in Figure 26a, the sensor’s front end may be attached to the user’s skin non-invasively. The sensor assay construction is shown schematically in Figure 26b. An open-face design of the front electrode allowed for the introduction of both fluids and lactate from sweat to be used in the construction of an assay and for lactate itself to be sensed.

**Figure 27:**
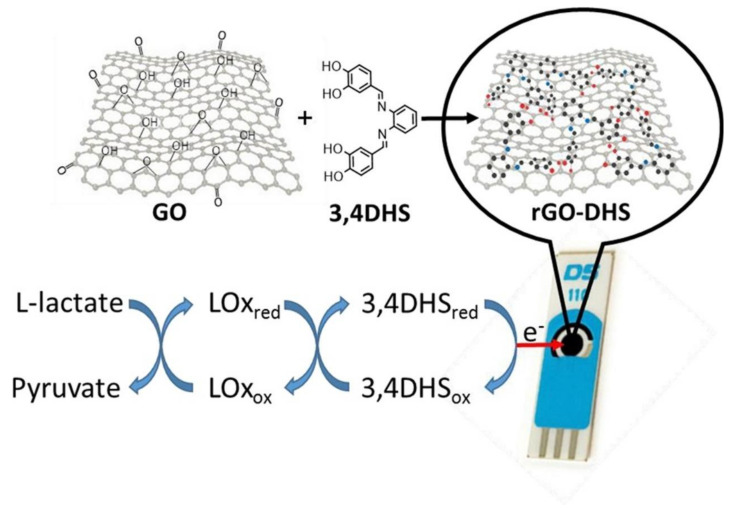
Scheme of the biosensing platform. (Reproduced from [558] with permission. Copyright 2015, Elsevier, Amsterdam, The Netherlands).

Schiff base ligand was used to concurrently reduce and alter graphene oxide (GO) utilising a one-step technique to create functionalized reduced graphene oxide, as reported in Bravo et al. [558]. The lactate oxidase (Lox) lactate biosensor was developed after spectroscopic and electrochemical investigation showed the presence of the characteristic hydroquinone/quinone moieties in the hybrid nanomaterial. With 2.9 μM detection limits, the resultant biosensing device has excellent analytical performance. Lactate biosensing is seen in Figure 27.

**Figure 28:**
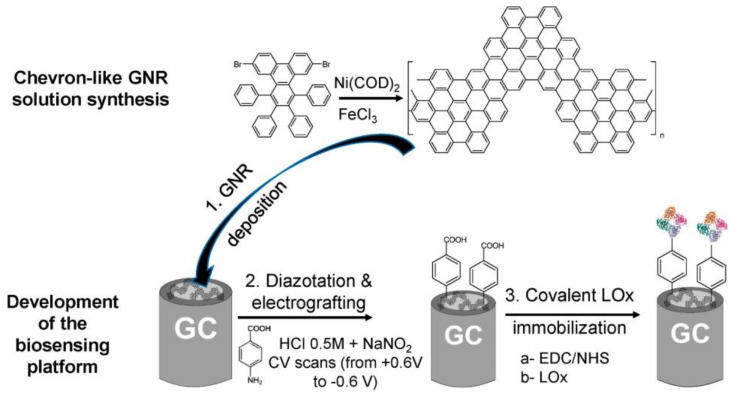
The creation of the lactate oxidase-based biosensor and the solution synthesis of the chevron−shaped GNR. (Reproduced from [559] with permission. Copyright 2022, Elsevier, Amsterdam, The Netherlands).

**Figure 29:**
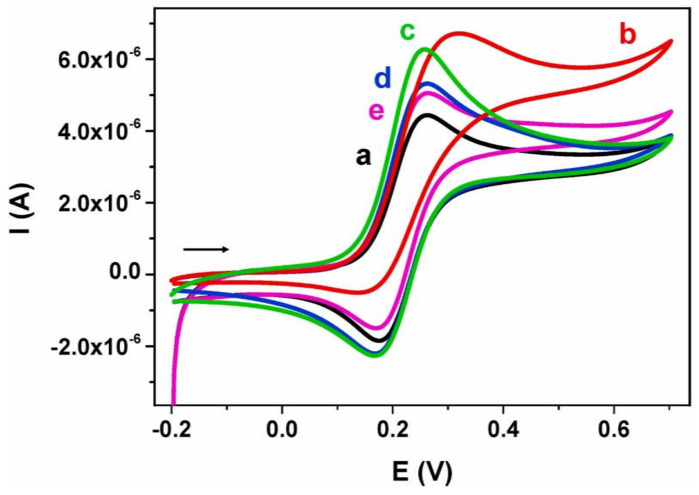
Cyclic voltammograms of Lactate in 0.1 M phosphate buffer with 1 mM HMF. (Reproduced from [559] with permission. Copyright 2022, Elsevier, Amsterdam, The Netherlands).

An electrochemical biosensor for lactate measurement has been devised and manufactured by Raquel Sainz et al. [559]. The enzyme Lox (lactate oxidase) is attached onto GNR (graphene nanoribbons), which were earlier manufactured by a solution-based chemical approach and employed as modifiers of GCE (glassy carbon electrodes), using a diazotation procedure. First, we grafted a 4-carboxyphenyl film onto the GNR-modified electrode surface by electrochemically reducing the matching 4-carboxyphenyl diazonium salt. We focused on the use of GNR in the manufacturing of the device and the use of a covalent strategy for enzyme immobilisation in our design of the electrochemical sensor, with the objective of enhancing the analytical capabilities of the resultant biosensor for the measurement of lactate. The immobilisation of the enzyme onto the transducer plays a critical role in the creation of biosensing platforms since it is critical that the enzyme maintains its full catalytic activity and stability following the immobilisation procedure. The enzyme (LOx) has been covalently connected to the carboxylic groups (COOH) of BzA (Figure 28), forcing it to arrange itself in such a manner that it can bind to the COOH groups exposed from the surface via its amino groups. This might have an impact on its active centre and catalytic activity. We measured the cyclic voltammetric response of the GCE/GNR/BzA/LOx system in a solution containing 1 mM of hydroxymethylferrocene in the absence (Figure 29, curve a) and presence of L−lactate to ensure that the enzyme retains its lactate recognition capability after being immobilised onto the modified electrode (Figure 29, curve b).

### 8.3. Uric Acid

**Figure 30:**
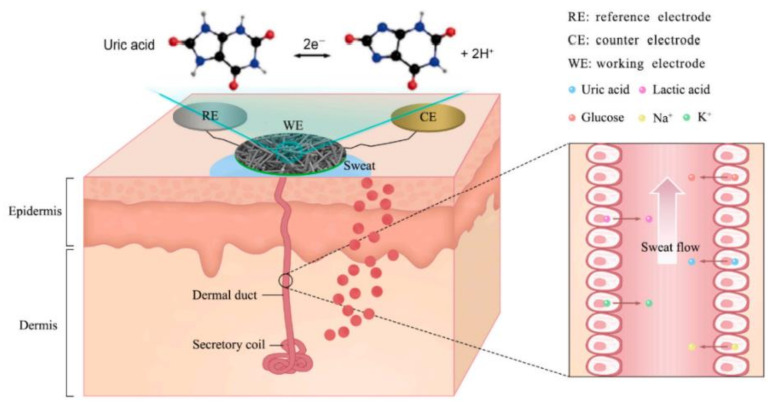
Illustrations showing the structure of the sweat glands, the release of biomarkers, and a wearable biosensor for detection of uric acid in sweat. (Reproduced from [563] with permission. Copyright 2021, Elsevier, Amsterdam, The Netherlands).

Sweat includes a wealth of physiological information that may represent a person’s current health status [560]. Figure 30 depicts the sweat gland anatomy and the non-invasive wearable sweat biosensor’s operating principle, which is enabled by a fibre structured sensing interface. There are a number of drawbacks to the sweat composition study of human health. Obtaining a big enough sample volume is one of the first hurdles to overcome. Additionally, the sweat biosensor’s sensitivity and reliability will be hampered by the evaporation and degradation of sweat chemical information throughout the testing procedure [561]. As a result, wearable sweat biosensors can detect and analyse biomarkers in sweat in real time with only a tiny quantity of sweat sample, which can address both of the aforementioned technical difficulties simultaneously. Detection of uric acid in sweat has received relatively little attention in the current sweat sensing landscape, with most investigations focusing on electrolyte (such as K^+^ and Na^+^) or glucose molecule alterations in sweat during exercise. According to clinical investigations, the amount of uric acid in sweat is strongly linked to the quantity of uric acid in blood [562]. Cardiovascular illness, renal disease, and metabolic diseases have all been linked to uric acid. More significantly, in clinical settings, uric acid is critical in the diagnosis of gout. As a result, it is important to use wearable biosensing equipment to measure the uric acid level in sweat in order to prevent and cure gout. A flexible and permeable sensor interface was developed to detect uric acid in sweat with high sensitivity. Electrically conductive and structurally flexible carbon nanofibers (CNFs) were created by heating electrospun polyacrylonitrile (PAN) nanofibrous membranes. Since CNFs are made from naturally occurring conductive materials, they do not need any further processing or fusion steps. A small diameter, porous structure, and high specific area of this CNFs-based electrode enable for better and quicker electron transport in electrochemical processes, as well as a more effective and appropriate contact between the reactants and the working electrode. A CNF-based wearable biosensor for sweat-based detection of uric acid has been described for the first time, according to the best of our knowledge. Table 6 shows the performance parameters of some good quality graphene based uric acid sensors available in the recent literature.

**Figure 31:**
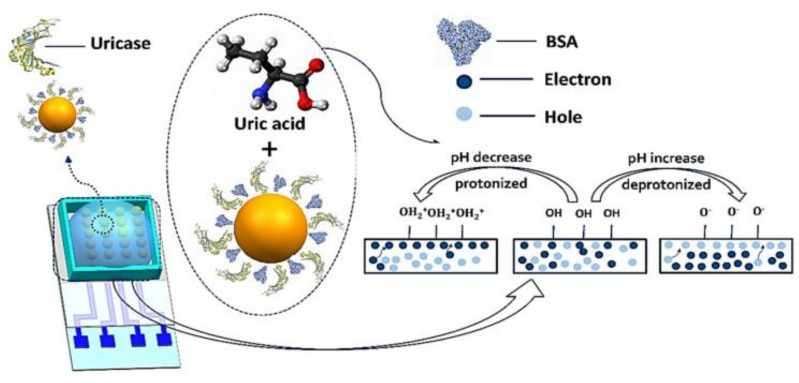
Working mechanism of graphene-based UA biosensor. (Reproduced from [564] under common creative.).

Figure 31 depicts the mechanism of our graphene-based biosensor for uric acid detection. During the catalytic reaction of uricase, uric acid is transformed to allantoin, carbon dioxide, and hydrogen peroxide, causing local pH alterations in the reaction channel. As the pH drops, hydroxyl groups on the channel’s surface may be protonated to OH^2+^ or deprotonated to O as the pH rises. As a result, OH^2+^ makes n-doped graphene, and O makes p-doped graphene, depending on the arrangement of the graphene/electrolyte interface. Doping holes or electrons into graphene may modify channel conductance, which is consistent with previous graphene research [564].

### 8.4. Detection of K^+^ and Na^+^

**Figure 32:**
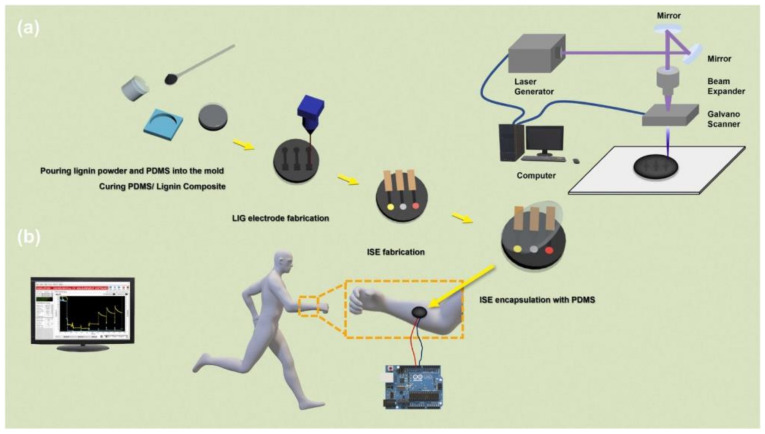
(**a**) Schematic representation of the LIG-based sensor platform’s manufacturing process. (**b**) An illustration of the sensor platform mounted to the wrist and the results of such sensing. (Reproduced from [576] under common creative.).

It is possible to collect data on a person’s health using sweat monitoring rather than blood sampling since sweat sampling may be performed directly on the skin. Other uses include diagnosing illnesses and other conditions. If wearable biosensors are to be widely used for longer monitoring of physiological data in addition to sweat sensing, they must include low-cost, high efficiency, multibiometric sensing-capability platforms. The authors of Chan-Woo Lee et al. [576] suggest a wearable multifunctional LIG sensor with Arduino-based readout circuits that measures sodium and potassium ion concentrations in human sweat at the same time. In Figure 32, we see an ion-selective electrode, which is an electrochemical sensor that measures the potential difference between a working electrode and a reference electrode in order to estimate the concentration of particular ions. The ionophore, plasticizer, lipophilic additive, and matrix formed the working electrode. The 2D CAD application was used to create a two-electrode approach with a working and a reference electrode for fabricating the LIG-based electrochemical potassium and sodium sensor (AutoCAD, San Rafael, CA, USA). In all, there were two ion sensor electrodes as well as a reference electrode in the device, with a 7 mm electrode diameter, a 2 × 10 mm electrode line width and length, and a 7 × 7 mm electrode pad size.

**Figure 33:**
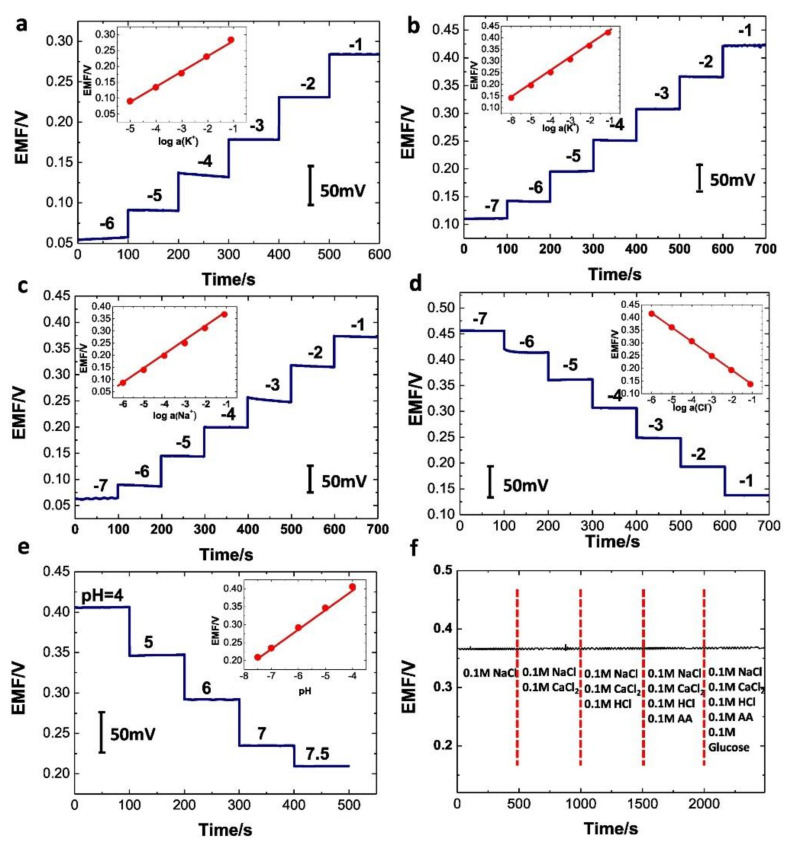
(**a**–**e**) The K^+^-GCE, K^+^-GPE, Na^+^-GPE, Cl^−^GPE, and H^+^-GPE calibration curves and open-circuit potential responses. (**f**) Interference testing for Na^+^-GPE. (Reproduced from [577] with permission. Copyright 2019, Elsevier, Amsterdam, The Netherlands).

Wearable potentiometric ion sensors, which monitor ion concentration levels in biofluids and offer information about a person’s health, have sparked a lot of interest. Significant advancements have been made, notably in the area of device integration. The critical interfacial water layer as well as the utilisation of transducer materials remain difficult to achieve. For the real-time study of sweat, QingboAn et al. [577] presented all-solid-state, a paper-based, flexible, ISE. To minimise the water-layer effect, a fluorinated alkyl silane was added to the superhydrophobic paper matrix. An excellent suspension of graphene with high capacitance and conductivity was used in the graphene-to-electron transducer to better stabilise the potential. There are four channels of integrated solid ISE that can concurrently monitor Cl^−^, K^+^, Na^+^ and pH. This wearable potentiometric sensor offers a high level of accuracy, strong potential stability, and low limits of detection, making it suitable for application in healthcare and clinical analysis. The potential responses of the ISE electrodes were investigated in solution containing the positive and negative ions of interest in various concentration from 10^−7^ to 1 M. (Figure 33). Figure 33a–e shows the calibration curves for the Cl-GPE, K^+^-GPE, Na^+^-GPE, K^+^-GCE, and H^+^-GPE. To give a baseline for comparison, we looked at the probable response of the K^+^-GCE. The linear range was found to be between 10^−1^ and 10^−5^ M, and the slope was found to be 49.4 ± 0.58 mV/decade (R^2^ = 0.9952) (Figure 33a). Since there is no transducer layer, the slope is lower than the Nernst response, which might lead to drift at low K^+^ concentrations. The slope rises to 52.0 ± 0.53 mV/decade (R_2_ = 0.9990) if the linear range is decreased from 10^−1^ to 10^−4^ M. The advantages of the graphene transducer are shown by the K^+^-GPE’s response slope, which is 57.0 ± 0.25 mV/decade (Figure 33b). Na^+^ and Cl^−^ were also evaluated since sweat contains a variety of ions, and the results showed that they had somewhat similar Nernstian slopes (Figure 33c,d). Sweat may be detected using the linear range, which is between 10^−1^ and 10^−6^ M. A Nernstian response is also seen in Figure 33e, which depicts the potential response time. The interference test for the Na^+^-GPE is shown in Figure 33f.

**Figure 34:**
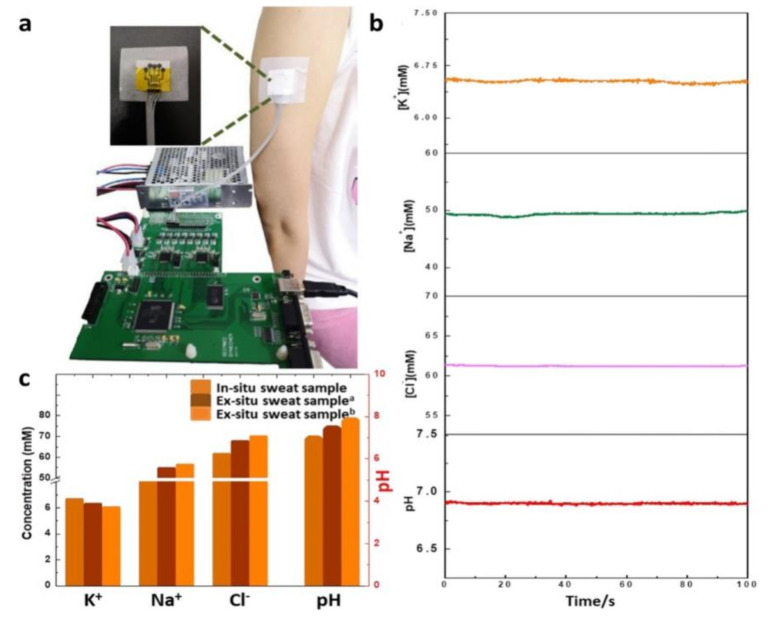
(**a**) An image of the detection instrument. (**b**) Results of in-sweat pH and target ion concentration real-time monitoring. (**c**) Sweat analysis findings in comparison to real-time test samples. (Reproduced from [577] with permission. Copyright 2019, Elsevier, Amsterdam, The Netherlands).

To track the ions in sweat, we enlisted the help of a volunteer who exercised for one hour. As illustrated in Figure 34a, the sensor was linked to a conductor that was connected to the Lab view system on the opposite side, allowing multichannel data to be communicated instantly. Figure 34b depicts real-time data corresponding to the levels of Cl^−^, Na^+^, K^+^ and pH present in the sweat. The indications are consistent. Following are the measurements made of the target ion concentrations: K^+^, Na^+^, Cl^−^ are 6.5, 49.5, and 61.4 mM and pH (6.91). Sweat was centrifuged and ICP-MS examined to provide a baseline for comparison (Figure 34c). K^+^ accuracy was determined to be 90%, Na^+^ accuracy to be 88.4%, Cl^−^ accuracy to be 87.6%, and pH accuracy to be 88.7%.

**Figure 35:**
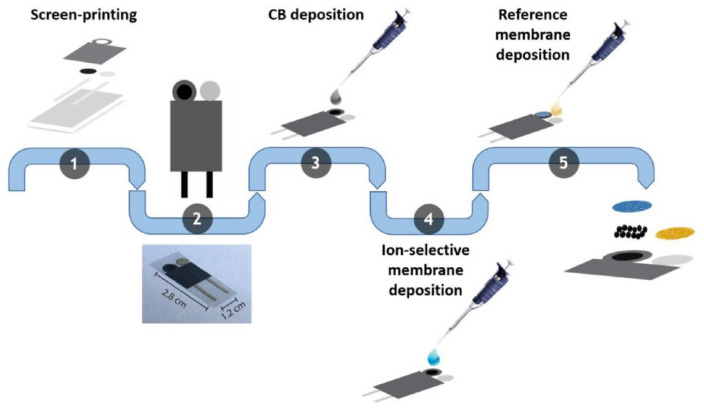
Diagram illustrating the many stages associated with producing modified SPEs. (Reproduced from [578] with permission. Copyright 2021, Elsevier, Amsterdam, The Netherlands).

It has been reported that Vincenzo Mazzaracchio et al. [578] developed new screen-printed electrodes that can be modified by carbon black and a polyvinyl butyral-based membrane that can be used to modify the reference electrode. The screen-printed electrochemical sensor revealed no aqueous layer formation between the working electrode and selective membrane after all parameters were optimised, as well as long-term stability, high shelf life, and resistance to light and oxygen interference. The sensor based on carbon black was able to detect sodium ions in the range of 10^−4^–10^−1^ M with a slope of 58 ± 3 mV/decade and 63 μM of detection limit. Three sweat samples taken during a running session were analysed, yielding concentrations of 47 ± 3, 55 ± 6 and 44 ± 4 mM, values consistent with the sodium ions present in healthy individuals. Artificial sweat was also used, with recovery rates of 90 ± 3%, 94 ± 2%, and 94 ± 5%. Figure 35 schematizes each phase, from screen printing through screen-printed electrodes (SPEs) modification.

**Figure 36:**
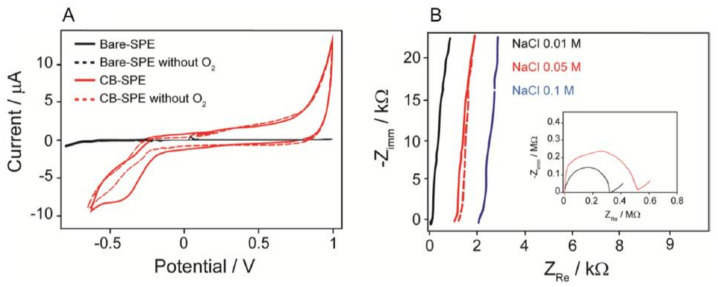
(**A**) Cyclic voltammetry was performed using 100 μL of 100 mM NaCl in the absence (dashed line) and presence (solid line) of oxygen, using Bare-SPE (black) and CB-modified SPE, with a scan rate of 25 mV/s. (**B**) Different NaCl concentrations were used to achieve electrochemical impedance spectroscopy using and CB-SPE. (Reproduced from [578] with permission. Copyright 2021, Elsevier, Amsterdam, The Netherlands).

In order to conduct a voltammetric experiment employing cyclic voltammetry in a solution of 0.1 M NaCl in the −0.8 V to 1 V potential range, Vincenzo Mazzaracchio et al. [578] used both bare (SPE) and CB-modified SPE (CB-SPE). Figure 36A (continuous line) shows that the capacitive current measured using the CB-SPE (red line) was greater than the capacitive current measured using the standard SPE (black line). In order to demonstrate the increase in capacitance current utilising the SPE modified with CB, a capacitive current of around 500 nA was calculated in the case of CB-SPE and 5 nA in the case of naked SPE at a 0 V. Figure 36B shows the Na^+^ ion selective membranes (Na + ISM) with CB-SPEs, and the impedance measurements of SPE/CB/Na + ISM and SPE/Na + ISM are displayed.

### 8.5. Other Analytes

For the study of human performance, the diagnosis of stress-related disorders, and the monitoring of mental health, it is essential to comprehend and evaluate the endocrine response to stress. The majority of current methods for stress monitoring rely on subjective questionnaires to achieve non-invasive, continuous, real-time stress measurement at the molecular level and prevent stress-inducing blood sample. Utilizing a wireless (integrated) device of sensing, Rebeca M. Torrente-Rodriguez et al. [579] examined the dynamics of the stress hormone cortisol in human sweat. A flexible sensor array that takes use of the remarkable electrochemical sensing capabilities of laser-induced graphene enables highly sensitive, focused, and effective cortisol detection. Torrente-Rodriguez et al. [579] described the first diurnal cycle of cortisol and a dynamic stress–response profile developed from human sweat. Our pilot investigation reveals an interesting opportunity for non-invasive dynamic stress monitoring using portable and wearable sensor devices by demonstrating a significant empirical connection between serum and sweat cortisol. Rapid, periodic monitoring and non-invasive detection of ethanol after intake have witnessed an increase in research in recent years. Disposable sensors, which are currently used in point-of-care and on-site detection systems, are inadequate for long-term monitoring. Julien Biscay et al. [580] have devised a low-cost, portable, and unique method for electrochemical real-time monitoring over many days. The sensor can exhibit ethanol oxidation in phosphate buffer and synthetic sweat by applying +0.9 V to the screen-printed electrode modified with polyaniline and utilising 1 mM ethanol as the average quantity of ethanol removed in sweat after ingesting one alcoholic beverage. Ethanol is detected using 0.1 M sodium bicarbonate and 50 measurements per day for 11 days by our enzyme-based electrochemical sensor. In this complex biological matrix, the sensor device demonstrates outstanding durability after three months of dry storage in an oxygen-free cabinet, even though quantitative data cannot be retrieved from it. The capacity to monitor complex biological matrices in real time is one of the fundamental problems for enzyme-based electrochemical sensors. Qualitative responses show how this sensor may be used by non-experts, which implies that the future generation of wearable electronics essential for alcohol monitoring might benefit from their broader applicability.

Powerful bioanalytical analytical techniques based on graphite and polymer-based electrochemical sensors have evolved in recent years. However, the majority of manufacturing techniques are not beneficial to the environment due to the usage of hazardous chemicals and the waste they create. Using graphite powder and thermal laminating sheets, Anderson A. Dias et al. [581] offered an alternate approach for producing flexible electrodes on plastic substrates without the use of chemicals that are hazardous. Electrodes created using the suggested method have shown flexibility, resilience, repeatability (a relative standard deviation of roughly 6%), and variety. All of the electrodes were evaluated using several techniques, including electrochemical impedance spectroscopy, cyclic voltammetry, and scanning electron microscopy. The electrode surfaces were bismuth-modified to demonstrate the notion by testing for zinc in perspiration. By actual sweat samples, the electrodes’ zinc concentrations were compared to those determined using atomic absorption spectroscopy and found to be statistically identical. A 3D-printed device was created to collect, store, and analyse the sweat sample in order to enable wearable applications. This device was connected with the suggested electrochemical system and secured to the abdomen using elastic tape. When the actual sample was spiked with various zinc levels for the matrix effect test, recovery values ranged between 85 and 106 percent, confirming the flexibility and resilience of the flexible electrodes created using the suggested manufacturing process.

## 9. Challenges

Non-enzymatic sensors have a long way to go in terms of commercialisation. In the majority of investigations, materialistic techniques based on a variety of substances and structural engineering have taken precedence. A practical sensor for detection of analytes from sweat cannot be developed using today’s experimental setups, which makes it evident that the systems and materials under consideration have significant functional constraints. These studies need a wide range of unique materials, which may provide new opportunities for breakthroughs in electrode and protective film technology. A more thorough investigation of the sensing mechanism is required for mass manufacturing, however, to identify the issues that prevent reliable functioning in clinical samples. It is common for diseases to develop slowly over time, with no outward symptoms or warning indications being visible to the individual affected. The commercialization of electrochemical biosensors, with the exception of diabetes, is still in its early stages. Glucose sensors are readily accessible in the market due to their widespread commercialization. Due to graphene-based transducers’ superior sensitivity and lesser cost, non-enzymatic glucose sensors are presently the most studied than enzymatic glucose sensors. Electronic biosensors are still in their infancy as a technology for detecting lung cancer, heart disease and asthmatic symptoms. The discovery of biomarkers for various diseases must be based on genuine samples/analytes. Simple solutions for biomolecule immobilisation and material functionalization may lead to practicality and commercialization at a lower cost. When used in the manufacture of transducers, nanomaterials incorporated with graphene-based materials have showed high performance in the detection of different analytes, namely the limit of detection in the region of ng/mL with greater selectivity and sensitivity. In order to achieve such high electrochemical performances, graphene nanosheets are uniformly distributed across a large surface area, allowing for fast electron transportation and an efficient immobilisation of bioreceptors. However, there are still a few obstacles that need to be overcome, such as the development of non-invasive electrochemical biosensors, a universal technique for the generation of graphene-based materials with the desired sizes and layers, the synthesis of a fine layer of graphene with zero defects, the best performing nanocomposite for electrode modification on diabetes detection, the best functionalization method for the development of immunosensors at a lower cost, and so on. There is now a focus on the point-of-care testing (POCT) market for biosensing platforms, where they provide significant advantages to healthcare providers and patients alike. POCT has a number of advantages, including the ability to be utilised at the patient’s bedside, quick detection, consistent results, ease of use, portability, and miniaturisation. From the selection of electrode materials, biological recognition element, chemicals preparation and immobilisation techniques, and 2D materials functionalization strategies, all these benefits may be attributed to the manufacturing procedures of nanodevices/biosensors. POCT is divided into two categories: tiny hand-held devices and big bench-top devices. The advancements in microfabrication and nanotechnology led to the development of the compact hand-held devices. In disease diagnostics, such compact hand-held devices also make it feasible to test the quality and quantity of a broad variety of analytes and to improve the diagnostic methods. As a result, disease identification processes may be streamlined by integrating biosensors and readout software with a real-time smartphone display. As a result, affordable POCT biosensors for routine health monitoring and management are now within reach of the majority of people. Health care facilities prefer non-invasive wearable sensors because they pose less danger and may continue to perform their functions even when they are removed from the body. Wearable technology has evolved from a novelty to a need in modern medicine. It will be a long time before medical devices can be made using these materials. Because of its potential to offer long-term authentic precise monitoring of tissues/organs/systems as well as support in diagnosis and therapy, implanted medical devices are progressively displacing conventional wearable and portable equipment. This is due to the advancement of materials and manufacturing techniques. Implantable devices, however, face a number of difficulties, including biocompatibility, biofouling, and power supply. Biodegradable and transient/biodegradable electronics have a wide range of implanted applications that can be destroyed without the need for further procedures or danger of infection. Biodegradable electronics for implants might use myeloperoxidase, an enzyme generated from human neutrophils, to break down the extensively scattered GO sheets. Non-invasive and invasive sensors used to monitor human health might be called an “augmented sense,” which extends the human senses. As a result, the development of a closed-loop illness management system that incorporates multifunctional sensors and feedback point-of-care treatment is critical. These gadgets, particularly implanted ones, need a source of power, especially in long-term use. An increasing trend in the construction of self-powered systems is the use of sensors and energy harvesting technologies, such as triboelectric nanogenerators (TENG), photovoltaics, radio frequency (RF), biofuel cells and thermoelectric. Finally, throughout the commercialization process, it is unavoidable to address issues of pricing and cost management. In order to produce vast quantities of graphene and graphene-based sensors at high throughputs, cost-effective and simple manufacturing techniques with outstanding uniformity need be devised. Materials have distinct benefits and disadvantages; thus, trade-offs must be made in order to meet the needs of various industries and applications. Although graphene has a wide range of unique properties, it also has limits. When it comes to biological applications, graphene’s zero gap structure has a low on/off ratio, which makes it less suitable for use. Functionalized organic molecules may be able to bridge the gap in its bandgap. Additionally, spintronics and strain-engineered lattice distortions have been tested. Due to its extreme sensitivity to environmental stimuli, graphene also lacks selectivity for target analytes. To enhance selectivity, one way is to add functional groups, bioreceptors, or a thin selective layer such as metal-organic frameworks to the surface (MOFs). Graphene’s moisture absorption and ultra-thinness also contribute to its poor long-term stability. In certain cases, a coating of stable thin layer materials may be all that is needed. The inherent characteristics of graphene may be readily (typically adversely) influenced by these material integrations, device construction, and processing stages when using graphene for functional devices in various applications. After addressing the most pressing issues, graphene-based technologies may go ahead with more confidence and success.

## 10. Future Outlook

Non-enzymatic sensors have sparked a lot of attention as an interesting substitute to overcome the inherent limits of sensors (enzymatic), and they are predicted to solve the stability problem as well as the difficult procedure for the mass production of sensors (enzymatic). As a result, each year sees an increase in the number of publications. Over the last ten years, rapid advances in nanotechnology and nanomaterials have fuelled the complexity and variety of this study field. The most notable result is significant advancement in applications (especially medical) of enzyme-free systems using electrodes having nanoporous characteristics [582]. It was described that film thickness, pore size variation, protective coating, and other parameters may result in non-enzymatic glucose sensors that can operate in undiluted human blood, plasma and serum. It was also explained that interferences from a variety of electroinactive and electroactive substances may be controlled. For non-enzymatic sensors to be commercialised, the next step in laboratory-scale experiments using novel materials must be clearly professional in order to produce mature technology. Exploring more characteristics of graphene-based materials helps to achieve greater selectivity, responsivity, reproducibility and stability towards sensing analytes from human sweat. Human healthcare has steadily evolved away from hospitals and into neighbourhoods (families, individuals). Because of this, sensors and technologies for health monitoring have received a lot of attention. Flexible electronics and sensors might benefit greatly from graphene’s chemical and physical capabilities. Both non-invasive flexible wearable sensors and invasive technologies have been included in this review of graphene sensors for human health monitoring. An array of vital signs and biomarkers can now be measured using graphene-based sensors, which has great promise for use in the healthcare industry, customised and preventative medicine, the treatment of illness and the development of human–machine interfaces. To enhance performance, new structures have been implemented, and the underlying sensing processes and technical breakthroughs have been extensively examined. Due to the widespread use of sensors and material science, a significant quantity of data will be created. These data will be used for anything from the Internet of Things to healthcare. This means that computational and statistical tools, such as machine learning, may be used to effectively analyse data and mine it. For effective data management, real-time data analytics skills are needed. Data collection, analysis, and storage, especially of personal health data, must be handled ethically and morally to ensure privacy. Graphene-based sensors have received a lot of attention recently, but there are still a number of scientific and technical obstacles that need to be overcome before any practical applications can be developed. Graphene and its derivatives must first be thoroughly tested for their effects on human health, including their biocompatibility and biological toxicity, as well as their influence on the environment. Graphene-based gadgets need to be thoroughly tested as well. Furthermore, robust sensing relies on biotic/abiotic interfaces that are conformal and functional. Gas and moisture sensors on the skin and other organs with permeability are needed. Multiple stimuli or very-low amount of biomarkers must be detected with good selectivity. If the sensor is incorporated into a multifunctional sensor, it may be responsive to stimuli that are not the intended stimuli. Environmental temperature fluctuation, for example, has an effect on the majority of sensor designs. Crosstalk may be present in integrated multifunctional sensors, which may concurrently or independently detect several signals. Mechanical durability and long-term stability are also required.

## 11. Conclusions

Sensors, especially wearable and implanted sensors, are key components of health monitoring systems and the interface to the human body because they can detect and analyse numerous analytes or signals with high sensitivity and specificity. Since graphene layers have a large specific surface area and an atomic thickness that allows complete carbon atoms to come into direct contact with analytes, graphene-based sensors offer higher sensitivity than silicon-based sensors. Graphene’s outstanding electrical conductivity and optical transparency also make it a perfect material for seeing bio-tissue in clear images with no distortions in visual appearance. Furthermore, due to graphene’s superior performance in biosensors, such as wide potential window, ease of functionalization, its large specific surface area, and high rate for transference of electron, receptors such as deoxyribonucleic acid, antibodies and enzymes can be efficiently immobilised on its surface. In this review, we have discussed most current graphene-based sensors, also including non-invasive and invasive health monitoring, along with their unique structures, sensing methodologies, and technological advances. Moreover, we included a comprehensive discussion about the methods for synthesis of graphene that is cost efficient, highly dependable, and scalable, as well as producing high yields and quality products. The properties of graphene such as mechanical, electrical and thermal properties are explained in detail. Graphene and its derivatives are known for their improved properties, and these have been synthesised to date due to the requirement of decreasing manufacturing costs, boosting product yield, obtaining superior final product stability, and enhancing sensing capabilities. However, the detailed knowledge about different analytes present in sweat along with the properties of a good sweat sensor is very important, and an elaborate discussion was made in separate sections. Graphene-based sensors have received a lot of attention recently, but there are still a number of scientific and technical obstacles that need to be overcome before any practical applications can be developed. Graphene and its derivatives must first be thoroughly tested for their effects on human health, including their biocompatibility and biological toxicity, as well as their influence on the environment. The future holds great opportunities for the development of efficient and advanced graphene-based sensors for the detection of analytes in sweat.

## Figures and Tables

**Table 1:** Mechanical characteristics of graphene and graphene-based nanocomposites.

Matrix	Synthesis Process	Filler	Fracture Strain (%)	Tensile Strength (MPa)	Reference
DGEBA	Three-roll mill	Thermally rGO	-	44.1 ± 5.0	[208]
Epoxy	In situ Polymerization (ISP)	Thermally rGO	5.0 ± 0.5	63 ± 1.0	[209]
WPU	ISP	rGO	-	14.6 ± 3.8	[210]
Phenol formaldehyde	ISP	rGO	-	1400 ± 0.04	[211]
PMMA	ISP	GO	-	66.8 ± 3.05	[212]
PI	ISP	FGO	6.9 ± 2.1	179.79 ± 17.72	[213]
Polybutadiene	ISP	Octadecylamine GO	450 ± 25	-	[214]
DGEBF	Resin transfer moulding	Sulfonic GO	-	41	[215]
PI	ISP	GO	8.5 ± 2.4	137.8 ± 9.7	[216]
Carboxylated Acrylonitrile butadiene rubber	Latex coagulation Method	GO	206 ± 16	8.8 ± 0.5	[217]
Polyvinylchloride (PVC)	Colloidal blending	GO	73.95	54.42	[218]
Polyamide 6	ISP	GO	-	64.9	[219]
DGEBA	ISP	GO	-	50	[220]
Epoxy	Casting	GO	55	13	[221]
PVA	Solution mixing	Graphene	19 ± 1	9.01 ± 0.3	[222]
WPU	Sol–gel	f-GNS	138 ± 30	20.2 ± 2.0	[223]
DETDA	Solution mixing	Diazonium-FG	3.9 ± 0.3	71.4 ± 0.8	[224]
Epoxy	Solution blending	GNP	3.61 ± 0.19	51.65 ± 1.43	[225]
Glassy epoxy	Solution blending	Graphite	4.75	60.76	[226]
Poly (lactic acid)	Melt compounding	GNP-small	10.9 ± 0.3	58.5 ± 0.7	[227]
PU	Solvent casting	GNP	5.7 ± 0.54	18.8 ± 1.95	[228]
Epoxy	Direct mixing method	GNPs	1.143	88.99	[229]
PP	Melt Compounding method	GNS	3.66 ± 0.75	30.16 ± 0.34	[230]
Epoxy	Solution mixing	Expanded graphene	25 (mm)	2.25	[231]
PVA	Solution mixing	Sulfonated graphene	-	97	[232]
Regenerated cellulose (RC)	Wet spinning	Graphene	-	360	[233]
PMMA	ISP	Graphene	1.79 ± 0.18	49.15 ± 0.86	[234]
Polypropylene (iPP)	Solution mixing	Graphene	2.9 ± 0.5	16 ± 3	[235]

**Table 2:** A study of the literature on the electrical conductivity of several graphene derivatives.

Material	Synthesis Method	Reducing Agent	Electrical Conductivity (S cm^−1^)	Reference
TrGO	Liquid Exfoliation (LE)	Thermal reduction	80	[247]
fGO	LE	Hydrazine and Pyrene groups	∼1000	[248]
GNS	LE	Hydrazine	24	[249]
rGO	LE	Hydroiodic acid and acetic acid	304	[249]
TrGO	LE	Thermal	727	[250]
TrGO	LE	Hydrazine and thermal annealing	298	[251]
GNS	LE	Ammonia and hydrazine	7.2	[252]
Gr	LE	Ammonia and hydrazine	5.5	[253]
TrGO	LE	Thermal	2.3	[254]
GNS	LE	Hydroquinone	-	[255]
Gr	LE	Hydrazine hydrate	1000	[256]
rGO	LE	-	72	[257]
rGO	LE	Dextrose	18	[258]
rGO	LE	Sodium borohydride	34	[258]
rGO	LE	Hydrobromic acid	36	[258]
rGO	LE	Hydrazine hydrate	58	[258]
rGO	LE	Hydroiodic acid	103	[258]
rGO	LE	KOH	60	[259]

**Table 3:** Thermal characteristics of nanocomposites made of graphene and modified graphene.

Matrix	Synthesis Process	Filler	CTE (°C)	k (W m^−1^ K^−1^)	Tg (°C)	Reference
DGEBA	Ball mill mixing	rGO	-	-	157.4 ± 1.8	[263]
DGEBA	Three-roll mill	rGO	-	-	154.8	[264]
PMMA	Solution blending	rGO	4.59 × 10^−5^	-	135.23	[265]
EP/GF	Hand lay-up process	Ethylenediamine (EDA)-FGO	-	-	127.9	[266]
Epoxy resin (CYD−128)	Solvent-free	Nanocrystal-f-GO	-	-	131.42	[267]
PVA	Casting method	GO	-	-	76	[268]
DGEBA	Polymerization	GO	-	-	71.5	[269]
Epoxy	Polymerization	Graphene-BN	-	6.2–9.5	-	[270]
EPON 862	Polymerization	Graphite	7.7 ± 0.1 × 10^−5^	1.0	135.3 ± 0.8	[271]
EPON 862	Solution blending	Exfoliated graphite	57.73 µm/(m °C)	5.0	-	[272]
Paraffin	Solvent evaporation	xGnPs	-	2.7	-	[273]

**Table 4:** Common detection techniques and sweat analysis.

Analytes	Recognition Component	Transduction Technique	Concentration in Sweat	References
Glucose	Glucose oxidase	Amperometry	10–200 µM	[281,282,283,284,285,286,287,288,289,290]
Lactate	Lactate Oxidase	Amperometry	5–20 mM	[282,283,284,285]
Uric acid	Uricase	Amperometry	2–10 mM	[292]
Cortisol	2D materials	Impedimetric sensor	8–140 ng mL^−1^	[296,297]
Ascorbic acid	Ascorbate oxidase	Amperometry	10–50 µM	[293,294,295]
Caffeine	Nanomaterials	Voltammetry	-	[310]
Tyrosine	Nanomaterials	Amperometry	6–240 µM	[298,299]
F17464	Carbon	Voltammetry	-	[301]
Ethyl glucuronide	Monoclonal antibody	Immunosensor	1.7–103 µg L^−1^	[300]
Cd^2+^	Bi	SWASV	<100 µg L^−1^	[316]
Zn^2+^	Bi	SWASV	100–1560 µg L^−1^	[315]
Ca^2+^	Ca ion selective electrode	Potentiometry	0.41–12.4 mM	[314]
NH^4+^	Nonactin ionophore	Potentiometry	0.1–1 mM	[313]
pH	Conducting polymer	Potentiometry	3–8	[310,311,312]
K^+^	K Ion selective membrane	Potentiometry	1–18.5 mM	[308]
Cl^−^	Ag/AgCl	Potentiometry	10–100 mM	[306,307]
Na+	Na Ion selective membrane	Potentiometry	10–100 mM	[300,301,302,303,304,305]

**Table 5:** Graphene-based glucose sensor.

Electrode	Sensitivity	Linear Range	Detection Limit	Reference
PtNW/RGO	56.11 μA mmol cm^−2^/L	0.032–1.89 mmol/L	4.6 μmol/L	[496]
Au–GO	25 μA mM^−1^ cm^−2^	0.05 mM–10 mM	-	[497]
AuNP-FLG	0.195 μA mM^−1^ cm^−2^	6 μM–28.5 mM	1 μM	[498]
Pt/GOH	137.4 μA mM^−1^ cm^−2^	-	-	[499]
AgNP-GO	11 μA mM^−1^ cm^−2^	1–14 mM	4 μM	[500]
AuNPs/GONR	59.1 μA mM^−1^ cm^−2^	0.005–4.92 mM	5 μM	[501]
Co/Fe/N-doped graphene	476.67 μA mM^−1^ cm^−2^	0–32.5 mM	37.7 μM	[502]
CoPC/graphene/IL/SPCE	-	0.01–13 mM	0.67 μM	[503]
HexagonalCo_3_O_4_/rGO	1.315 mA mM^−1^ cm^−2^	-	0.4 μM	[504]
CuO nanoflakes/rGO	53.5 μA mM^−1^ cm^−2^	1–2000 μM	0.19 μM	[505]
PDDAgraphene/CuO nanocomposite	4982.2 μA mM^−1^ cm^−2^	0.4–4000 μM	0.2 μM	[506]
CuNCs/graphene	4532.2 μA mM^−1^ cm^−2^	25 μM–4 mM	250 nM	[507]
CuO/rGO	2221 μA mM^−1^ cm^−2^	0.4 μM–12 mM	0.1 μM	[508]
SnO_2_/rGO	1.93 AM^−1^ cm^−2^	50 μM–500 μM	13.35 μM	[509]
NiNPs/graphene	8652 μA mM^−1^ cm^−2^	5–550 μM	1.85 μM	[510]
Cu(OH)_2_/PGF	3.36 mA mM^−1^ cm^−2^	1.2 μM–6 mM	1.2 μM	[511]
NiFe/GO	173 μA mM^−1^ cm^−2^	0.05–5 mM	9 μM	[512]
PtNi alloy/graphene glassy electrode	40.17 μA mM^−1^ cm^−2^	0.5–40 mM	0.355 μM	[513]
CoNi_2_Se_4_/rGO	18.89 mA mM^−1^ cm^−2^	1 μM–4.0 mM	0.65 μM	[514]
Cu/Ni/graphene/Ta	17 857 μA mM^−1^ cm^−2^	0.24–2.33 mM	0.0027 μM	[515]
Co_3_O_4_NF/GOHs	492.8 A mM^−1^ cm^−2^	0.25 mM–10 mM	-	[516]
NiCoS_2_/rGO	1753 μA mM^−1^ cm^−2^	0.001–5 mM	0.078 μM	[517]
Pd–CuO/rGO/SPE	3355 μA mM^−1^ cm^−2^	6 μM–22 mM	30 nM	[518]
Pd/NiO@Nile-rGO	-	0.020–20.0 mmol L^−1^	2.2 μmol L−1	[519]
Pt–CuO/rGO	3577 μA mM^−1^ cm^−2^	Up to 12 mM	0.01 μM	[520]
PtPd-IL-rGO	1.47 μA mM^−1^ cm^−2^	0.1–22 mM	2 μM	[521]
Pt-Ni/graphene	30.32 μA mM^−1^ cm^−2^	0.5–20 mM	2 μM	[522]
PdCu/GE	48 μA mM^−1^ cm^−2^	up to 18 mM	20 μM	[523]
NiO/Pt/ERGO	668.2 μA mM^−1^ cm^−2^	0.05–5.66 mM	0.2 μM	[524]
PtPdNCs/GNs	1.4 μA mM^−1^ cm^−2^	Up to 24.5 mM	-	[525]
Ni–Co/rGO	1773.6 μA mM^−1^ cm^−2^	0.01–2.65 mM	3.79 μM	[526]
CuONPs/sulphur-doped graphene	1298.6 μA mM^−1^ cm^−2^	0.1–10.5 mM	80 nM	[527]
MnO_3_O_4_/N-doped rGO	0.026 μA μM^−1^ cm^−2^	1.0–329.5 μM	0.5 μM	[528]
MnO_3_O_4_/N-doped graphene/CPE	0.1011 μA μM^−1^ cm^−2^	2.5–529.5 μM	1.0 μM	[529]
CuNiO/N-doped graphene	7.49 μA mM^−1^ cm^−2^	0.2 μM–0.3 mM	50 nM	[530]
Cu/N-doped graphene	43.13 μA mM^−1^ cm^−2^	0.004–4.5 mM	1.3 μM	[531]
MnO_2_/CuO/GO	-	0.55–4.4 mM	53 μM	[532]
NiNPs/PEDOT/rGO/GCE	36.15 μA μM^−1^ cm^−2^	1 μM–5.1 mM	0.8 μM	[533]
Cu–Co/CS/rGO/GCE	1921 μA μM^−1^ cm^−2^	0.015–6.96 mM	10 μM	[534]
CuNPs/PAA/Graphene	-	0.0003–0.6 mM	0.08 μM	[535]
NiNPs/CS/rGO	318.4 μ AμM^−1^ cm^−2^	Up to 9 mM	4.1 μM	[536]
PdNPs/Nafion/Graphene	-	10 μM–5 mM	1 μM	[537]

**Table 6:** Graphene-based uric acid sensor.

Materials	LoDuM	Range M	Reference
Fe_3_O_4_/SiO_2_/GO	0.07	0.5 × 10^−6^–2.5 ×10^−4^	[565]
MoS_2_−rGO	3.8	4.0 × 10^−6^–4.0 × 10^−5^	[566]
GOx−chitosan/Co_3_O_4_/Au	0.1	3.0 × 10^−7^–3.0 × 10^−6^	[567]
ZnO−graphene	5.0	5.0 × 10^−6^–8.0 × 10^−5^	[568]
CeO_2−x_/C/rGO	2.0	4.98× 10^−5^–1.05 × 10^−3^	[569]
Au/Pd−rGO	5.0	2.0 × 10^−8^–5.0 × 10^−4^	[570]
rGO−ZnO	0.33	1.0 × 10^−6^–7.0 × 10^−5^	[571]
Graphene/Neutral Red	0.076	0.5 × 10^−6^–2.0 × 10^−2^	[572]
Polytetraphenylporphyrin/PPy/GO	1.15	5.0 × 10^−6^–2.0×10^−4^	[573]
Au/ZnO/PPy/rGO	0.09	1.0 × 10^−6^–6.8 × 10^−6^	[574]
ZnO/PANI/rGO	0.042	1.0 × 10^−9^–1.0 × 10^−7^	[575]

## Data Availability

Not applicable.
